# Inhalable Plant Exosome-like Nanoparticles from *Houttuynia cordata* Ameliorate Acute Lung Injury via Redox Modulation and Immune Reprogramming

**DOI:** 10.3390/pharmaceutics18101235

**Published:** 2026-09-29

**Authors:** Ting Yin, Junjian Hu, Lijuan Xu, Shichen Huang, Bao Zhang, Long Qiu, Yanlei Liu, Daxiang Cui, Zhengjie Zhou, Jincheng Zeng, Menglei Zha

**Affiliations:** 1 Research Center of Nano Technology and Application Engineering, The First Dongguan Affiliated Hospital, School of Pharmacy, Guangdong Medical University, Dongguan 523808, China; wsyt132@163.com (T.Y.); ah18475532913@163.com (J.H.);; 2 Dongguan Research Center for Biomedical Nano Engineering Technology Research, Dongguan 523808, China; 3 Dongguan Key Laboratory of Medical Bioactive Molecule Development and Translational Research, Guangdong Provincial Key Laboratory of Medical Immunology and Molecular Diagnostics, Guangdong Medical University, Dongguan 523808, China; 4 Dongguan Key Laboratory of Chronic Inflammatory Diseases, The First Dongguan Affiliated Hospital, School of Pharmacy, Guangdong Medical University, Dongguan 523710, China

**Keywords:** noninvasive pulmonary delivery, plant-derived exosome-like nanoparticles, redox modulation, acute lung injury, immune microenvironment

## Abstract

**Background**: Acute lung injury (ALI) features oxidative stress, macrophage dysfunction, and barrier disruption, with limited safe therapies. Plant-derived exosome-like nanovesicles (PENs) delivered noninvasively to the lung are promising but understudied. **Methods**: In vitro, HELNs were evaluated for their ability to scavenge ROS, restore mitochondrial membrane potential (MMP) modulate M1/M2 polarization, and suppress pro-inflammatory cytokines. RNA-seq was also used to evaluate the TNF/NOD-like pathway and mitochondrial-related gene expression. In vivo, the evaluated parameters included biodistribution, therapeutic outcomes (lung wet/dry weight ratio, BALF protein concentration, MPO and MDA levels, histology), immune cell profiles, and cytokines. Biosafety was assessed via hematology, serum biochemistry, and histology. **Results**: In vitro, HELNs acted dose-dependently by scavenging ROS, recovering MMP, promoting M2 and suppressing M1 polarization, and reducing TNF-α, IL-6 release. Additionally, RNA-seq confirmed downregulation of TNF/NOD-like signals and regulated mitochondrial gene expression. In vivo, nebulized HELNs preferentially accumulated in inflamed lungs, with significantly higher pulmonary retention than intravenous delivery. HELNs reduced lung wet/dry weight ratio, BALF protein, MPO and MDA levels, and histological injury scores; decreased neutrophils and CD4^+^/CD8^+^ T cells; increased alveolar macrophages and Tregs; and lowered TNF-α, IL-1β, IL-6. No significant systemic toxicity was observed. **Conclusions**: Nebulized HELNs offer a safe, effective, noninvasive ALI therapy by disrupting the oxidative stress-inflammation vicious cycles, reprogramming pulmonary immunity, and restoring barrier integrity, with strong translational potential.

## 1. Introduction

Acute lung injury (ALI) and acute respiratory distress syndrome (ARDS) are critical pulmonary diseases characterized by three hallmarks: a potent inflammatory cascade, diffuse alveolar-capillary barrier injury, and noncardiogenic edema, all of which lead to severe hypoxemia and respiratory failure [1]. Globally, ALI/ARDS accounts for approximately 10% of intensive care unit admissions and has a mortality rate of 30% to 46%, representing a major cause of death among critically ill patients [2]. 

Pathogenesis starts with lung injury (direct or indirect), leading to a cytokine storm defined by excessive pro-inflammatory cytokines and Reactive Oxygen Species (ROS) [3]. ROS amplify inflammatory signals, drive immune cell infiltration, and promote the polarization of macrophages toward the pro-inflammatory M1 phenotype, ultimately forming a vicious cycle [4,5]. These M1 macrophages secrete large quantities of cytokines (e.g., tumor necrosis factor-α (TNF-α), interleukin-1 beta (IL-1β), and interleukin-6 (IL-6)), further exacerbating inflammation [6]. This intense inflammatory response not only degrades tight junction proteins (e.g., Zonula Occludens-1 (ZO-1) and occludin) and directly disrupts the alveolar-capillary barrier but also induces the apoptosis of epithelial and endothelial cells, ultimately leading to the accumulation of protein-rich edema fluid within the alveolar spaces [7]. Current clinical management remains predominantly supportive, including lung-protective ventilation, restrictive fluid management, and glucocorticoids such as dexamethasone [8]. However, current pharmacological options are non-specific, are accompanied with major adverse effects (e.g., immunosuppression, metabolic disturbances), and do not improve long-term outcomes [9]. Therefore, novel therapeutic strategies are urgently needed to directly target inflamed lung tissue, suppress the inflammatory cascade, mitigate oxidative injury, and promote tissue repair.

Nanoparticle-based drug delivery systems offer a novel strategy for the treatment of ALI, providing advantages such as enhanced drug stability and controlled release [10]. However, many synthetic nanomaterials face challenges in clinical translation, including immunogenicity, systemic toxicity, and complex preparation processes [11,12]. As alternatives, mammalian-derived extracellular vesicles (EVs) have attracted attention for their biocompatibility, anti-inflammatory properties, and ability to modulate complex biological pathways [13]. 

For example, extracellular vesicles originating from mesenchymal stem cells have been proven capable of exerting reparative effects in lung injury models [14]; however, their widespread use is limited by high production expenses, poor isolation efficiency, and inconsistent batch quality. Recently, plant-derived exosome-like nanoparticles (PENs) have emerged as promising biotherapeutics that overcome the scalability limitations of animal-derived EVs [15]. PENs isolated from edible plants offer excellent biocompatibility, high yield, and low cost [16]. Notably, PENs are enriched with lipids, proteins, and metabolites from their parent plants, conferring intrinsic anti-inflammatory, antioxidant, and immunomodulatory properties [17,18]. PENs derived from ginger, grapefruit, and lemon have demonstrated therapeutic efficacy in treating diseases such as colitis and rheumatoid arthritis [19]. Given these characteristics, PENs have strong potential for interrupting the self-perpetuating loop of oxidative stress and inflammation during ALI development. Despite these advantages, the use of PENs for noninvasive pulmonary delivery in ALI treatment has rarely been explored.

The route of administration critically influences the therapeutic efficacy of treatments for pulmonary diseases. Systemic delivery often results in inadequate lung drug concentrations, leading to off-target accumulation in other organs and potentially significant systemic toxicity [20]. In contrast, pulmonary delivery via nebulization enables direct delivery of lung tissue, thereby maximizing local bioavailability while minimizing systemic side effects [21]. This noninvasive approach is preferred for treating respiratory diseases and is particularly relevant for ALI, where the delivery system must penetrate the viscous mucus barrier characteristic of inflamed airways to reach the deeper lung parenchyma and be efficiently taken up by inflammatory cells [22]. In this context, nebulization of PENs represents an innovative yet underexplored strategy. The intrinsic stability and optimal size range of PENs suggest their potential to effectively traverse pulmonary biological barriers, while their natural origin may reduce airway irritation. Together, these features position PENs as a safe and efficient platform for targeted ALI therapy.

*Houttuynia cordata*, commonly known as the heartleaf Houttuynia herb, is a well-recognized medicinal and edible plant in East Asia. It is traditionally valued for its heat-clearing, detoxifying, and anti-inflammatory properties, and is regarded in traditional Chinese medicine as a plant with broad-spectrum infection-fighting capabilities [23]. Inspired by these attributes, we hypothesized that PENs derived from fresh *Houttuynia cordata* (HELNs), when delivered via nebulization, could be effective novel therapies for ALI.

**Scheme 1:**
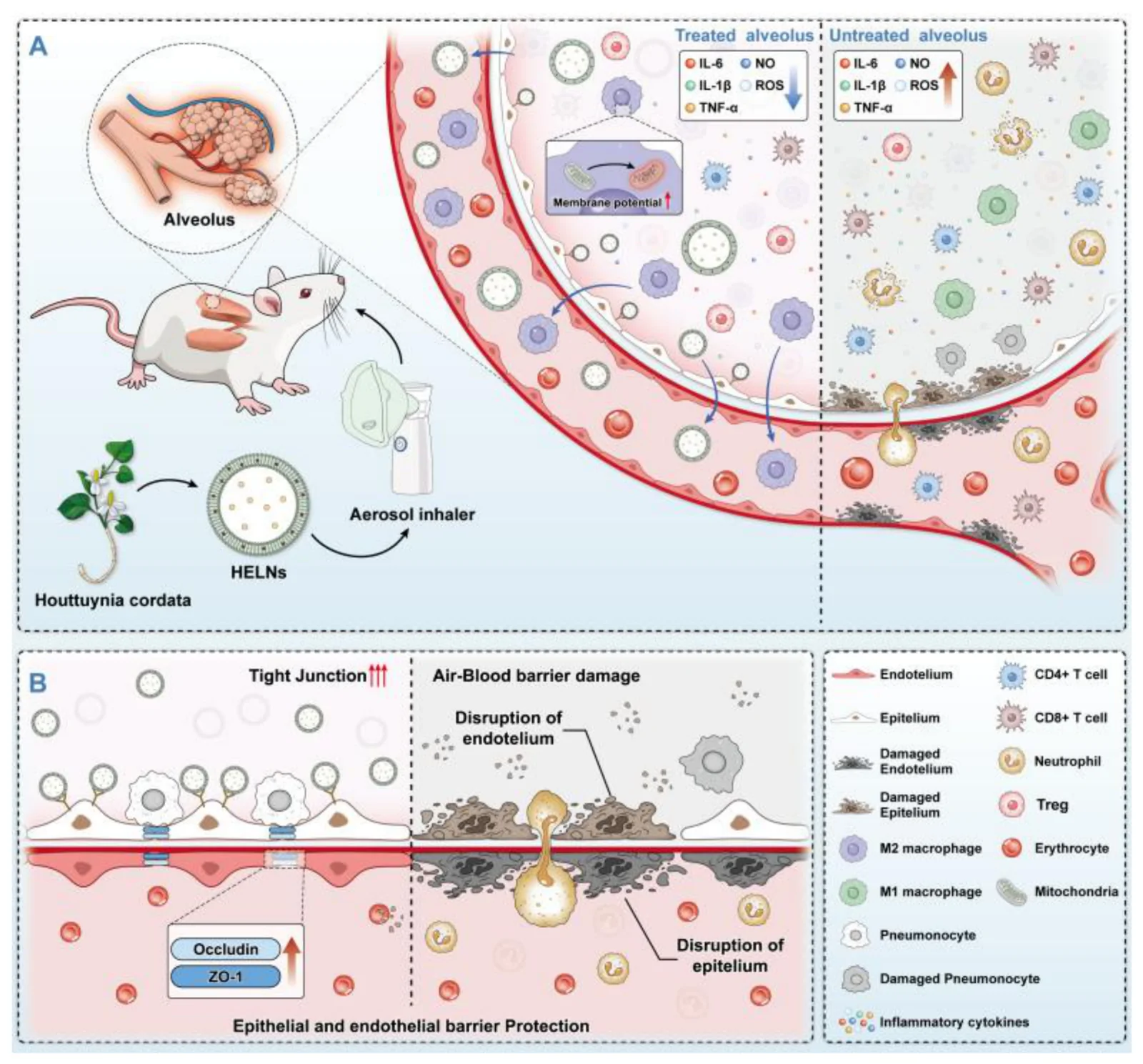
Preparation of HELNs and their therapeutic effects on ALI. (**A**) *Houttuynia cordata* exosome-like nanoparticles (HELNs) are isolated and administered via nebulized inhalation. In vivo, HELNs potently suppress inflammatory mediators, alleviate pulmonary inflammation, reprogram immunity responses, and ameliorate lung tissue injury. (**B**) HELNs exert the therapeutic outcome in LPS-induced ALI through their anti-inflammatory activity and protection of the pulmonary epithelial and vascular endothelial barriers.

In this study, we report that nebulized HELNs derived from fresh *Houttuynia cordata* serve as an effective therapy for ALI (Scheme 1). We successfully isolated and characterized HELNs and investigated their therapeutic potential in a murine model of LPS-induced ALI. Mechanistically, HELNs exert dual protective effects through redox modulation and immune reprogramming, which collectively alleviate pulmonary inflammation and restore alveolar–capillary barrier integrity. Overall, this study identifies nebulized HELNs as a safe, effective, and scalable therapeutic approach for ALI, suggesting promising prospects for the clinical management of ALI/ARDS and other inflammatory pulmonary disorders.

## 2. Materials and Methods

### 2.1. Materials and Instruments

The aerial parts of *Houttuynia cordata* were collected from Zengcheng (Guangzhou, China), in August 2024. This plant was identified by Professor Zeng Jincheng according to Flora of China. DCFH-DA and dexamethasone acetate were obtained from Aladdin (Shanghai, China). The JC-1 kit and lipopolysaccharide (LPS) were obtained from Solarbio (Beijing, China). Cell Counting Kit 8 (CCK-8), nitric oxide (NO), Fast Silver Stain Kit, Cy5.5, bicinchoninic acid (BCA) protein assay, and Dil cell membrane labeling were acquired from Beyotime (Shanghai, China). Mouse IL-6 ELISA kit, TNF-α ELISA kit and IL-1β ELISA kit utilized in this work were sourced from Jiangsu Meimian industrial Co., Ltd. (Yancheng, China). The myeloperoxidase (MPO) assay kit, which uses a colorimetric method, and the malondialdehyde (MDA) assay kit, which employs the TBA method, were obtained from Nanjing Jiancheng (Nanjing, China). The antibodies for flow cytometry were obtained from Biolegend (San Diego, CA, USA).

Ultracentrifuge (Optima XPN-100 Ultracentrifuge, A99846, Beckman Coulter Inc., Brea, CA, USA); nanoparticle tracking analyzer (NanoSight NS300, Spectris Instrument Systems Co., Ltd., Shanghai, China); Zeta potential and dynamic light scattering analyzer (Zetasizer Nano ZS90, Malvern Panalytical Ltd., Great Malvern, UK); confocal microscope (TCS SP8 Laser Scanning Confocal Microscope, Leica Microsystems, Wetzlar, Germany); flow cytometer (FongCyte™ Flow Cytometer, C2060, Beijing Challenbio Biotechnology Co., Ltd., Beijing, China); and microplate reader (Spark Multimode Microplate Reader, SPARK, Tecan Group AG, Männedorf, Switzerland).

### 2.2. Animal Studies

All animal experiments were approved by the Animal Ethics Committee of Guangdong Medical University (Approval No. GDMU-2025-000151). Specific pathogen-free (SPF) male BALB/c mice (6–8 weeks old), weighing 18–22 g, were purchased from Guangdong Zhiyuan Biomedical Technology Co., Ltd. (Guangzhou, China).

### 2.3. Preparation and Characterization of HELNs for Their Physicochemical Properties and Aerosolization Performance

#### 2.3.1. Extraction and Purification of HELNs

Fresh *Houttuynia cordata* tissues were initially washed with tap water to eliminate soil residues, followed by multiple rinses with sterile water. The tissues were then sectioned into small fragments, homogenized using a tissue grinder, mixed with phosphate-buffered saline (PBS), and filtered through gauze to collect the tissue extract. The crude extract was subjected to sequential differential centrifugation at 4 °C: 3000× *g* for 20 min, 6000× *g* for 30 min, and 9000× *g* for 30 min to remove cell debris and large vesicles. The resulting supernatant was further ultra-centrifuged at 100,000× *g* for 90 min to obtain the pellet. The pellet was resuspended in PBS, followed by filtration through a 0.22 μm membrane filter to yield purified HELNs. The prepared HELNs were stored at 4 °C for short-term preservation or at −80 °C for long-term storage.

#### 2.3.2. Characterization of HELNs

Transmission electron microscope (TEM) imaging was carried out on a JEM-1400Plus transmission electron microscope supplied by JEOL (Tokyo, Japan). The nanoparticle concentration and size distribution were evaluated with a NanoSight NS 300 (Spectris Instrument Systems Co., Ltd., Shanghai, China). Zeta potential, particle size, and PDI were measured at 25 °C using a NanoZS 90 instrument (Malvern Panalytical Ltd., Malvern, UK). The protein equivalent was determined using the BCA method, and 1 μg of protein corresponds to 5.3 × 10^10^ particles. HELNs at concentrations of 6.25 μg/mL, 12.5 μg/mL and 25 μg/mL were denoted as HELNs-L, HELNs-M and HELNs-H, respectively.

#### 2.3.3. Silver Staining

Samples were lysed on ice by vortexing in prechilled lysis buffer containing protease inhibitors. After high-speed centrifugation at 4 °C for 15–20 min, the supernatant was collected as total protein. Denatured proteins were separated by 10% sodium dodecyl sulfate-polyacrylamide gel electrophoresis (SDS-PAGE) and silver-stained per the manufacturer’s protocol.

#### 2.3.4. Determination of Aerosol Droplet Size Distribution

Aerosol droplet size distribution was measured at the nebulizer outlet by laser diffraction (Malvern Panalytical, Malvern, UK). The measurement range was set to 0.1–2000 μm, and the nebulizer was positioned to allow the spray plume to fully cross the laser beam. Data acquisition was performed at a sampling frequency of 2.5 kHz for 30–60 s per measurement, with three replicates per sample. Volume median diameter (VMD, Dv50), Dv10, Dv90, and span (Dv90 − Dv10)/Dv50 were calculated using the Spraytec software. Ambient temperature and humidity were recorded before and after each measurement to ensure consistent conditions.

#### 2.3.5. Determination of the Output Rate and Nebulization Efficiency of the Nebulizer

Nebulization efficiency was determined by gravimetric analysis combined with BCA protein quantification. Briefly, the initial total mass of the nebulizer reservoir and the formulation (m_1_) was precisely weighed using an analytical balance. Nebulization was initiated with simultaneous timing, and the total nebulization time (T) was recorded when the aerosol stream was visibly interrupted. The residual mass of the nebulizer and its contents (m_2_) was then weighed, and the mass of liquid consumed during nebulization (Δm) was calculated as Δm = m_1_ − m_2_. The aerosol output rate (g/min) was calculated as Δm/T. All measurements were performed in triplicate.

Nebulization efficiency was further evaluated by comparing the total protein content of HELNs loaded into the nebulizer (M_0_ = protein concentration before nebulization × formulation volume) with the residual protein retained in the system after nebulization. The residual protein was collected by thoroughly rinsing the nebulizer reservoir, tubing, and mixing chamber with PBS, and the total volume of the rinsing solution was recorded. The protein concentration in the rinsing solution was quantified using the BCA method, and the total residual protein mass (M_1_ = C_1_ × V_1_) was calculated. Nebulization efficiency was then calculated as: Nebulization efficiency (%) = (M_0_ − M_1_)/M_0_ × 100%.

#### 2.3.6. Characterization of the Physicochemical Properties of HELNs Before and After Nebulization

The pre-nebulization HELN stock solution (Before) was characterized for particle size and zeta potential by dynamic light scattering (DLS), and protein concentration was determined by the BCA method. Post-nebulization samples (After) were collected via a condensation tube (ice-bath) placed between the nebulizer outlet and the exposure port under the same nebulization conditions as formal administration. Approximately 1–2 mL of the aerosol condensate was collected and analyzed for the same parameters using identical methods. All measurements were performed in triplicate at 25 °C.

#### 2.3.7. Triton X-100 Lysis Assay

Purified HELNs were incubated with various concentrations (0.01%, 0.025%, 0.05%, and 0.1%, *v*/*v*) of Triton X-100 in PBS at room temperature for 30 min. The particle concentrations before and after detergent treatment were measured using a nanoparticle tracking analyzer. The membrane lysis rate was calculated as follows:

Lysis rate (%) = [(C_0_ − C_1_)/C_0_] × 100%

where C_0_ and C_1_ are the particle concentrations (particles/mL) before and after Triton X-100 treatment, respectively. All measurements were performed in triplicate.

### 2.4. Metabolomic Sequencing Analysis

For metabolomics analysis, metabolites were extracted by organic solvent protein precipitation. Ultra-performance Liquid Chromatography (UPLC) tandem mass spectrometry (MS/MS) in positive/negative ion mode was used to acquire precursor-ion mass spectrum (MS1) and product-ion mass spectrum (MS2) data. Raw data were converted to mzML (MSConvert) and processed with XCMS software for peak alignment, extraction, and area integration, yielding retention times, m/z values, and intensities per ion. After quality control (QC) filtering, the metabolites were identified and relatively quantified by matching MS1 to Human Metabolome Database (HMDB) (https://hmdb.ca/, 26 April 2026) and MS/MS to an in house library.

### 2.5. In Vitro Cellular Experiments

#### 2.5.1. Hemolysis Assay

Healthy mouse blood was collected into anticoagulant tubes and mixed. Red blood cells (RBCs) were obtained via low-temperature centrifugation, washed with cold saline, and diluted to 4%. The RBCs suspension was incubated with HELNs (6.25–100 μg/mL) at 37 °C, and then centrifuged, and the supernatant absorbance was measured at 540 nm using a microplate reader. The hemolysis rate was calculated as follows:

Hemolysis rate = (Test sample OD − Negative control OD)/(Positive control OD − Negative control OD) × 100%

#### 2.5.2. Cell Culture

L929, C166, and RAW264.7 cells (Zishan Biotechnology, Wuhan, China) were cultured in Dulbecco’s Modified Eagle Medium with high-glucose (DMEM) supplemented with 10% fetal bovine serum (FBS) and 1% penicillin-streptomycin under standard culture conditions (37 °C, 5% CO_2_).

#### 2.5.3. In Vitro Biocompatibility Assessment

RAW264.7 cells were seeded at 5 × 10^4^ cells per well and allowed to reach an appropriate density. The cells were then treated with HELNs at serial concentrations of 6.3, 12.5, 25, 50 and 100 μg/mL, and cell viability was assessed via the CCK-8 assay. Cell viability (%) = (OD treated − OD blank)/(OD untreated − OD blank) × 100%. The blank wells contained only culture medium and CCK-8 solution, while the control wells contained RAW264.7 cells without HELNs treatment.

#### 2.5.4. Uptake of HELNs by RAW264.7 Cells

HELNs were labeled with Dil dye by incubation for 30 min in the dark, and then ultracentrifuged to remove free dye. Overnight-cultured RAW264.7 cells were washed with PBS and co-incubated with Dil-HELNs for 1, 2, 4, 8 and 12 h. After fixation with 4% paraformaldehyde (PFA) and DAPI staining, the cells were imaged via confocal microscopy. The same protocol was used for flow cytometric quantification of HELNs uptake.

#### 2.5.5. In Vitro Measurement of NO and Pro-Inflammatory Cytokine Release Levels

RAW264.7 cells were cultured overnight in dishes, after which the medium was replaced with fresh medium containing graded concentrations of HELNs. After incubation, the supernatant was collected, and NO/pro-inflammatory cytokine levels were measured to evaluate HELNs-mediated promotion or inhibition of their secretion.

#### 2.5.6. Regulation of RAW264.7 Cell Polarization

RAW264.7 cells (8 × 10^5^/well) were seeded in six-well plates overnight, washed with PBS, and then treated with LPS for a defined time to induce polarization. After 24 h of co-incubation with various HELNs concentrations, the cells were harvested and stained with antibodies under dark conditions. Flow cytometry was then applied to determine the regulatory effect of HELNs on macrophage polarization.

#### 2.5.7. In Vitro Antioxidant Activity

RAW264.7 cells were first incubated with LPS for inflammatory induction, followed by 24 h treatment with PBS or graded concentrations of HELNs. The medium was then replaced with DCFH-DA solution, and the cells were further incubated. After washing with PBS to remove unbound dye, the intracellular ROS levels were determined via flow cytometry. ROS detection by confocal microscopy followed the same steps as above.

#### 2.5.8. Mitochondrial Damage Assessment

The MMP was measured using the JC-1 assay to assess the effects of HELNs. Briefly, RAW264.7 cells (8 × 10^5^/well) were seeded in confocal dishes and cultured overnight. After LPS stimulation, the cells were co-incubated with various concentrations of HELNs for 24 h. Staining was performed according to the JC-1 kit protocol, followed by assessment of changes in the membrane potential via confocal microscopy and flow cytometry.

#### 2.5.9. RNA-Seq Analysis of RAW264.7 Cells

RAW264.7 cells were cultured in six-well plates and divided into two groups: the PBS group (LPS + PBS) and the HELNs group (LPS + HELNs), with a treatment duration of 24 h. Total RNA was extracted, and RNA integrity was assessed using a bioanalyzer, followed by RNA sequencing (with three biological replicates per condition). Through bioinformatics analysis, differentially expressed genes were defined as those exhibiting a ≥1.5-fold change.

### 2.6. In Vivo Animal Experiments

#### 2.6.1. LPS Challenge Mouse Model of Acute Lung Injury (ALI)

Specific-pathogen-free (SPF) male BALB/c mice were acclimated for one week prior to being randomly assigned to five groups: normal control, LPS-induced disease model control, LPS + low-dose HELNs (target exposure dose:0.5 mg/mouse), LPS + high-dose HELNs (target exposure dose:1 mg/mouse),and LPS + dexamethasone (target exposure dose:0.4 mg/mouse). Except for the normal control group, all the mice received intratracheal instillation of LPS (20 μL, 5 mg/kg) to induce ALI. Mice were challenged with LPS at 0 h, followed by the first treatment at 4 h and the second treatment at 24 h post-LPS. All animals were euthanized at 48 h post-LPS for sample collection. Throughout the trial, general health was monitored and recorded daily.

#### 2.6.2. Lung Wet/Dry Weight Measurement

The lung wet/dry (W/D) ratio was used to assess pulmonary edema. At 24 h post-treatment, the lungs were collected, rinsed with PBS, blotted, weighed (wet weight), oven-dried, and reweighed (dry weight). W/D = wet weight/dry weight.

#### 2.6.3. Determination of Protein Concentration in Bronchoalveolar Lavage Fluid (BALF)

The protein concentration in BALF serves as an indicator of enhanced permeability of the alveolar-capillary barrier. Following euthanasia, bronchoalveolar lavage was promptly conducted. The BALF was centrifuged, the supernatant was collected, and the protein concentration was quantified via BCA.

#### 2.6.4. Measurement of MPO and MDA Concentrations in Lung Tissue Homogenates

Mouse lung tissues were homogenized, and the homogenate was centrifuged at low temperature (manufacturer’s instructions). The supernatant was collected for MPO and MDA measurements.

#### 2.6.5. DHE and TUNEL in Lung Tissue 

To evaluate oxidative stress levels in lung tissue, dihydroethidium (DHE) staining was employed to detect ROS. Frozen tissue sections were rewarmed, washed with PBS, and incubated with DHE at 37 °C in the dark. Following washing with PBS, the nuclei were counterstained with DAPI, and the sections were mounted for imaging via a confocal microscope. Terminal deoxynucleotidyl transferase-mediated dUTP nick-end labeling (TUNEL) staining was used to assess apoptosis. The sections were deparaffinized, rehydrated, proteinase K-treated, and then incubated with TUNEL mix (dark). After being washed with PBS and stained with DAPI, the slides were mounted and imaged via fluorescence microscopy for quantification.

#### 2.6.6. Imaging of ZO-1 and Occludin Expression in Lung Tissue

The tight junction proteins ZO-1 and occludin were stained by immunofluorescence to assess lung barrier integrity. The paraffin-embedded sections were subjected to citrate based antigen retrieval, blocked (3% bovine serum albumin (BSA), 1 h, room temperature (RT)), and incubated overnight with primary antibodies at 4 °C. After washing, the sections were incubated with fluorescent secondary IgG (1 h, RT, dark), counterstained with DAPI, mounted, and imaged via confocal microscopy.

#### 2.6.7. H&E Staining

Harvested lung tissue specimens were fixed in 4% formaldehyde solution for 48 h. After fixation, the samples were subjected to gradient dehydration in 70%, 80% and 90% ethanol for 30 min at each concentration, followed by two dehydration treatments with 95% and 100% ethanol, 20 min per treatment. The dehydrated tissues were cleared in xylene and embedded in paraffin to produce 5 μm-thick tissue sections. The sections were stained with hematoxylin-eosin (H&E), and observation and image analysis were performed using K-viewer software and semi-quantitative assessment was conducted according to the modified Mikawa scoring system.

### 2.7. Immunocyte Profiling and Inflammatory Cytokine Measurement

#### 2.7.1. Flow Cytometric Analysis of Immune Cells in BALF

The BALF was centrifuged to collect the supernatant, and the pellet was re-suspended with ice-cold FBS. The collected cells were stained with FITC-Ly6G/APC-CD11b, and neutrophils (Ly6G^+^CD11b^+^) were quantified via flow cytometry. For alveolar macrophages, FITC-Siglec-F/APC-CD11c staining was used, and Siglec-F^+^CD11c^+^ cells were enumerated.

#### 2.7.2. Flow Cytometric Analysis of Immune Cells in Lung Tissue

To assess the regulatory impact of HELNs on macrophage polarization, single-cell suspensions derived from lung tissue were stained with FITC-F4/80 and PE-CD86 antibodies at 4 °C for 30 min under dark conditions. Following washing and resuspension, the percentage of F4/80^+^CD86^+^ M1 macrophages was quantified via flow cytometry. Similarly, the cells were stained with FITC-F4/80 and PE-CD206 antibodies to determine the proportion of F4/80^+^CD206^+^ M2 macrophages.

To evaluate the local T cell immune equilibrium within lung tissue, single-cell suspensions were stained with CD4-APC (1:500), CD8-FITC (1:500), and CD3-PE (1:500) to identify CD8^+^ (CD3^+^CD8^+^) and CD4^+^ (CD3^+^CD4^+^) T cells. The obtained single-cell suspension from lung tissues was placed into flow cytometry tubes. The cells were stained with CD4-FITC (1:500) and CD25-APC (1:500), mixed, and incubated at 4 °C in the dark for 30 min. After centrifugation and washing, the cells were successively treated with fixation buffer and permeabilization buffer for fixation and permeabilization. Following resuspension, Foxp3-PE (1:500) was added and mixed, and the cells were incubated at room temperature for 30 min. After centrifugation, the cells were resuspended and ready for flow cytometric analysis. Flow cytometry was performed on a FongCyte™ Flow Cytometer(Beijing Challenbio Biotechnology Co., Ltd., Beijing, China), and the data were analyzed with FlowJo 10.9.0.

#### 2.7.3. Measurement of Inflammatory Cytokines in BALF

BALF cytokine levels (TNF-α, IL-1β, and IL-6) were determined via ELISA (kit protocol). After incubation and washing, the wells were reacted with biotin-antibody and HRP-streptavidin, and then with the substrate. The absorbance at 450 nm was read, and the concentrations were derived from standard curves.

### 2.8. Pulmonary Deposition and Biodistribution Study

For comprehensive visual and quantitative assessment of lung delivery and in vivo transport of nebulized HELNs, HELNs were coupled with the near-infrared fluorescent dye Cy5.5. Cy5.5 NHS ester was added to the HELNs suspension and incubated at room temperature for 30 min in the dark with gentle shaking every 15 min. The reaction was terminated by adding Tris-HCl (pH 8.0) and further incubated for 10 min in the dark to quench unreacted NHS ester. Free dyes were removed by ultracentrifugation at 100,000× *g* for 1.5 h at 4 °C. The supernatant was discarded, and the pellet was washed with PBS. This washing step was repeated 2–3 times until the supernatant became clear. The purified Cy5.5-HELNs were resuspended in PBS and stored at 4 °C in the dark.

Mice were divided into an LPS-induced model group and a PBS control group. On the day after modeling, DiI@HELNs (200 µg protein per mouse) were administered via nebulization using a homemade oral-nasal exposure system for 10 min. At 1, 3, 6, and 12 h after nebulization, the animals were euthanized, and the heart, liver, spleen, lungs, and kidneys were harvested for ex vivo fluorescence imaging (IVIS). An intravenous injection control group (*n* = 3 per group) was included, receiving the same dose via the tail vein, for comparison with pulmonary accumulation at 3 h after nebulization. The total fluorescence intensity of the region of interest in each organ was quantitatively analyzed.

### 2.9. In Vivo Biosafety Assessment of HELNs

To assess the in vivo biosafety of HELNs, SPF male BALB/c mice (6–8 weeks old) were exposed to nebulized inhalation for three consecutive days. At the end of the treatment period, peripheral blood samples were collected for serum biochemistry and hematological parameters analyses. Liver/kidney function indicators including alanine transaminase (ALT), aspartate transaminase (AST), total bilirubin (TBIL), blood urea nitrogen (BUN), and creatinine (CRE), as well as blood counts parameters including red blood cells (RBC), white blood cells (WBC), and hemoglobin (HGB) were measured to rule out systemic metabolic issues. The major organs (heart, liver, spleen, lung, and kidney) were also paraffin-embedded and H&E-stained. Histomorphological alterations were semi-quantitatively evaluated via a histopathological scoring system. Furthermore, TUNEL staining was conducted on lung tissue to detect apoptosis, providing further confirmation of pulmonary safety.

### 2.10. Statistical Analysis

The data are shown as the means ± SDs. GraphPad Prism 9.3.0 was used for statistical analysis. *t*-tests (two groups) and one-way ANOVA (multiple groups) were applied. When the ANOVA results indicated significant differences among the groups, Tukey’s multiple comparisons test was employed as the post hoc method for pairwise comparisons. Significance was accepted at *p* < 0.05. * *p* < 0.05, ** *p* < 0.01, and *** *p* < 0.001, with “ns” denoting no significant difference.

## 3. Results

### 3.1. Isolation and Characterization of HELNs

**Figure 1:**
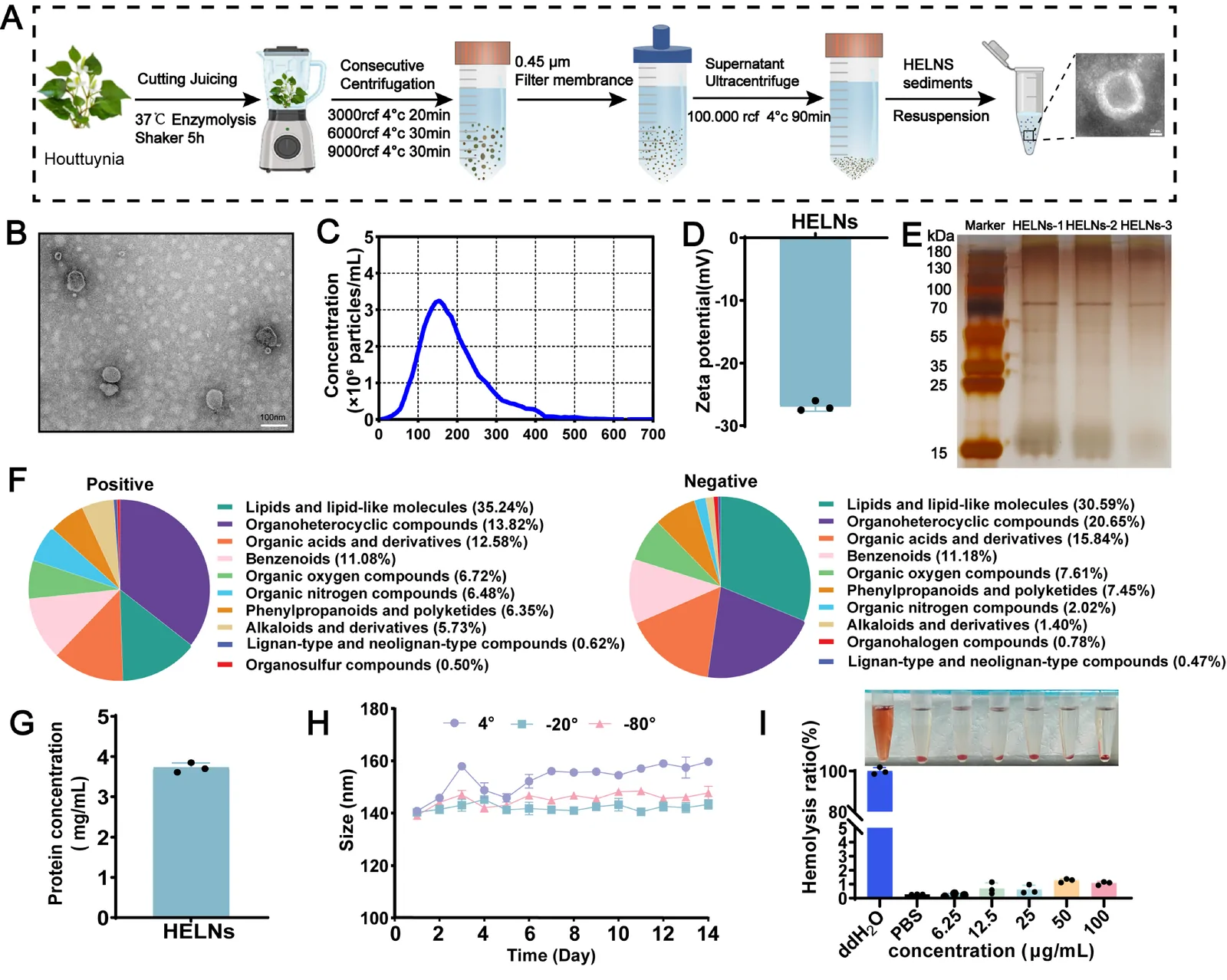
Preparation and characterization of HELNs. (**A**) Schematic of HELNs preparation by differential centrifugation. (**B**) Transmission electron microscopy (TEM) image of HELNs, scale bar: 100 nm. (**C**) Nanoparticle tracking analysis (NTA) of HELNs particle size and concentration. (**D**) Zeta potential distribution of HELNs. (**E**) Silver-stained 10% SDS-PAGE gel showing HELNs proteins at varying concentrations. (**F**) Metabolites in HELNs were classified based on charge: “Positive charge” denotes metabolites with a positive charge, while “Negative charge” denotes those with a negative charge. (**G**) The protein concentration of HELNs was measured via the BCA method. (**H**) Particle size analysis of HELNs under different storage conditions (*n* = 3). (**I**) Hemolytic activity of HELNs at various concentrations (*n* = 3).

Ultracentrifugation is a widely employed technique for the extraction and purification of PENs from plant sources [24]. HELNs were successfully isolated from *Houttuynia cordata* using stepwise centrifugation (3000–100,000× *g*). Fresh Houttuynia herb (*Houttuynia cordata*) from the heartleaf was rinsed, sectioned, and homogenized to obtain juice. Low speed centrifugation (3000× *g* for 20 min, then 6000× *g* for 30 min) was first performed to remove plant fibers and large debris, followed by medium speed centrifugation (9000× *g* for 30 min) to eliminate cell debris and intact organelles. HELNs were ultimately isolated through a high-speed centrifugation step (100,000× *g* for 1.5 h) as depicted in Figure 1A. Characterization of HELNs by TEM, dynamic light scattering (DLS), nanoparticle tracking analysis (NTA), gel electrophoresis, and SDS-PAGE revealed that they are spherical or oval vesicles with a classic bilayer membrane and a mean diameter of 90 nm (Figure 1B), which is consistent with typical PENs. NTA revealed an average particle size of 151.7 nm and a high particle concentration of 3.0 × 10^9^ particles/mL (Figure 1C). The discrepancy in average particle size between TEM and NTA measurements is attributed to the complete dehydration of HELNs during TEM analysis, in contrast to their swollen state during NTA measurement [25]. Meanwhile, the Triton X-100 lysis assay showed that approximately 77% of the particles were susceptible to lysis by 0.1% Triton X-100, supporting the membrane-enclosed nature of the majority of the isolated HELNs (Figure S1C). Zeta potential analysis revealed a value of −26.9 mV (Figure 1D), suggesting good stability and dispersibility.

To determine the molecular weight distribution of HELNs proteins, SDS-PAGE was performed, revealing a range of approximately 15–180 kDa (Figure 1E). As shown in Figure 1F, metabolomic profiling of HELNs indicates that plant exosomes mainly consist of lipids and lipid-like molecules constituting the membrane skeleton, along with abundant metabolites such as organic acids and their derivatives, benzenoids, and organic heterocyclic compounds. This reflects their multiple functions in intercellular communication, stress response, and defense regulation. Furthermore, complementary detection via positive- and negative-ion methods revealed that plant exosomes are enriched in lipids and organic acids simultaneously, positioning them as important nanocarriers in plant stress adaptation and signal transduction. To quantify HELNs for subsequent experiments, the protein concentration was measured via a bicinchoninic acid (BCA) assay with a standard curve (Figure S1A), yielding a concentration of 3.72 mg/mL (Figure 1G). The final protein equivalent obtained from 1 kg of fresh whole *Houttuynia cordata* plants was approximately 23.81 mg. The storage stability of HELNs was then examined. HELNs were stored at various temperatures, and the particle size remained stable at −80 °C for 14 days. Storage at either −20 °C or −80 °C was considered relatively stable, as illustrated in Figure 1H and Figure S17. Finally, the hemolytic activity of HELNs was assessed. Red blood cells were exposed to various concentrations of HELNs (6.25, 12.5, 25, 50, 100 µg/mL), and the findings indicated low hemolytic activity at all the tested concentrations (Figure 1I).

To evaluate the feasibility of precise pulmonary delivery of HELNs via nebulization, this study employed a mesh nebulizer as the experimental device (Figure S3A) and systematically characterized its aerosolization behavior and key delivery performance. First, droplet size distribution measurements revealed that the volume median diameter (VMD) of the nebulized droplets was 2.83 μm, with 73.85% of the droplets having a diameter of less than 5 μm. Meanwhile, the proportion of inhalable particles with a diameter of less than 10 μm was as high as 96.07%, while that of fine particles with a diameter of less than 2 μm accounted for 24.09% (Figure S3B), indicating that this nebulization system can generate aerosol particles with favorable inhalable characteristics and possess the aerodynamic potential for deposition in the lower respiratory tract. On this basis, batch-to-batch consistency was further evaluated by gravimetric analysis, and the results showed that the nebulization time, output efficiency, and output rate for the three batches of HELNs were (2.33 ± 0.08) min/mL, (79.52 ± 1.74)%, and (0.43 ± 0.02) g/min, respectively (Figure S3C–E), demonstrating that the nebulization efficiency and delivery uniformity were consistent across different batches. Notably, comparison of the physicochemical properties of the nanoformulations before and after nebulization (Figure S3F–I) revealed that the particle size increased from 156.7 nm to 270.7 nm, the PDI increased from 0.251 to 0.447, and the Zeta potential decreased from −26.0 mV to −18.6 mV, whereas the protein concentration remained essentially unchanged (439.73 μg/mL vs. 449.32 μg/mL). This result indicates that although the shear force during nebulization induced a certain degree of nanoparticle aggregation, the particle size remained within the submicron range and no significant loss of protein content was observed; therefore, the stability of HELNs under this nebulization condition is considered acceptable. In summary, HELNs exhibits favorable performance in terms of aerodynamic particle size distribution, fine particle fraction, delivery consistency, and nebulization stability, providing a solid formulation foundation for pulmonary delivery via nebulized inhalation.

### 3.2. Uptake of HELNs by Macrophages and Their Anti-Inflammatory Activity In Vitro

**Figure 2:**
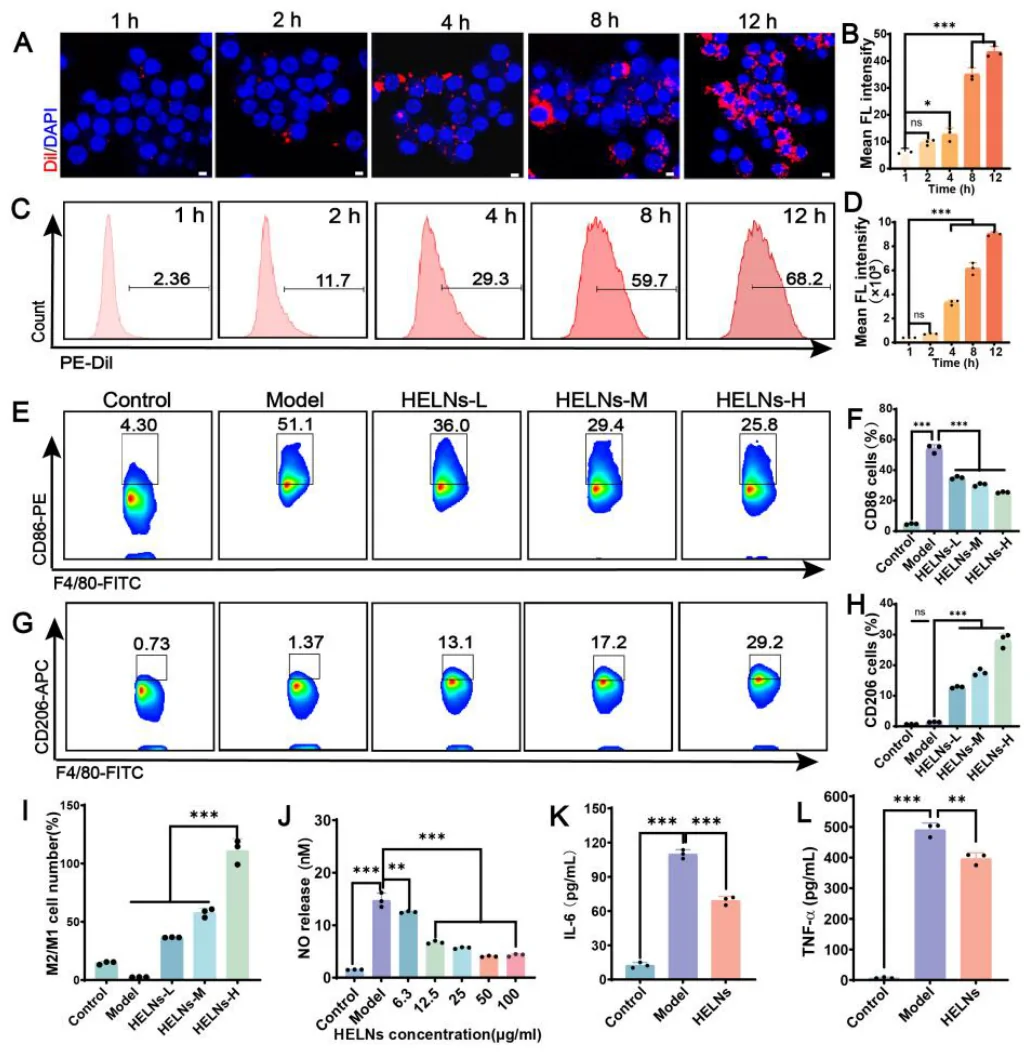
Uptake of HELNs by macrophages and their anti-inflammatory activity in vitro. (**A**) Confocal microscopy images and (**B**) semiquantitative analysis of RAW264.7 cells uptake Dil-labeled HELNs at 1, 2, 4, 8, and 12 h. Scale bar: 5 μm. (**C**) Flow cytometry analysis of Dil-labeled HELNs uptake by RAW264.7 cells at the specified time points, and (**D**) corresponding semiquantitative analysis. (**E**) Flow cytometric analysis of the expression of CD86, a marker indicative of M1 macrophages, in different treatment groups, and (**F**) semiquantitative analysis. (**G**) Flow cytometric analysis of the expression of CD206, a marker of M2 macrophages, in various treatment groups, and (**H**) semiquantitative analysis. (**I**) The ratio of M1 to M2 macrophages in different treatment groups. (**J**) Effects of HELNs on LPS-induced NO release in RAW264.7 cells. The impact of HELNs on the LPS-induced release of TNF-α (**K**) and IL-6 (**L**) in RAW264.7 cells. Statistical significance: * *p* < 0.05, ** *p* < 0.01, *** *p* < 0.001, ns means no significant; *n* = 3.

The uptake kinetics were investigated by coculturing Dil-labeled HELNs with RAW264.7 cells for 1, 2, 4, 8, and 12 h to evaluate the time-dependent uptake process. Confocal microscopy analysis (Figure 2A,B) revealed a significant increase in the red Dil fluorescence signal with increasing incubation period, indicating effective uptake of HELNs. This observation was corroborated by quantitative flow cytometry analysis, where the histogram (Figure 2C) revealed a rightward shift in the positive cell population with prolonged incubation, indicating increased fluorescence intensity. Statistical analysis (Figure 2D) revealed that, after 12 h of incubation, the uptake rate of HELNs reached 68.2%. These findings indicate that HELNs are efficiently take up by macrophages in a time-dependent manner, which is expected to regulate their phenotype and function.

Dysregulated macrophage polarization is a critical step in the progression of inflammatory diseases [26]. The modulatory effect of HELNs on LPS-induced M1/M2 polarization was confirmed by flow cytometric detection of the M1 marker CD86, which was significantly decreased following HELNs treatment. M1 macrophage numbers were significantly greater in the LPS group than in the control group (Figure 2E), confirming that LPS induced pro-inflammatory polarization. HELNs dose-dependently reduced the percentage of CD86 positive cells, an effect most pronounced at the highest tested concentration of 25 μg/mL (HELNs-H) (Figure 2F). In contrast, LPS markedly reduced the expression of CD206, a marker of M2 macrophages (Figure 2G), whereas HELNs treatment dose-dependently increased the proportion of CD206-positive cells (Figure 2H). Analysis of the M2/M1 ratio (Figure 2I) revealed a marked decrease following LPS stimulation, an effect that was dose-dependently rescued by HELNs. Thus, HELNs promote the phenotypic switching of macrophages from M1 to M2. To further assess the anti-inflammatory effects of HELNs, the release of key inflammatory mediators was measured in LPS-treated RAW264.7 cells. HELNs significantly reduced LPS-induced NO overproduction in a concentration-dependent manner (Figure 2J). The NO levels were quantified via a standard curve (Figure S2). Based on the NO release experiment, CCK-8 assays were conducted on RAW264.7, C166 and L929 cells with various concentrations of HELNs. HELNs did not exhibit apparent cytotoxic effects on RAW264.7, C166 or L929 cells at therapeutic concentrations. (Figure S4A–C). Quantitative analysis of pro-inflammatory cytokines via ELISA was then performed to further explore the anti-inflammatory properties of HELNs. The concentrations of IL-6 and TNF-α were quantified by constructing standard curves (Figure S5A,B). The results revealed that the secretion levels of the pro-inflammatory cytokines IL-6 (Figure 2K) and TNF-α (Figure 2L) were significantly greater in the LPS model group than in the control group; following treatment with HELNs, the secretion levels of both IL-6 and TNF-α were significantly reduced, indicating that HELNs can effectively inhibit the LPS-induced release of pro-inflammatory factors from macrophages and exert an anti-inflammatory effect.

### 3.3. Role of HELNs in Scavenging Reactive Oxygen Species and Restoring Mitochondrial Membrane Potential

**Figure 3:**
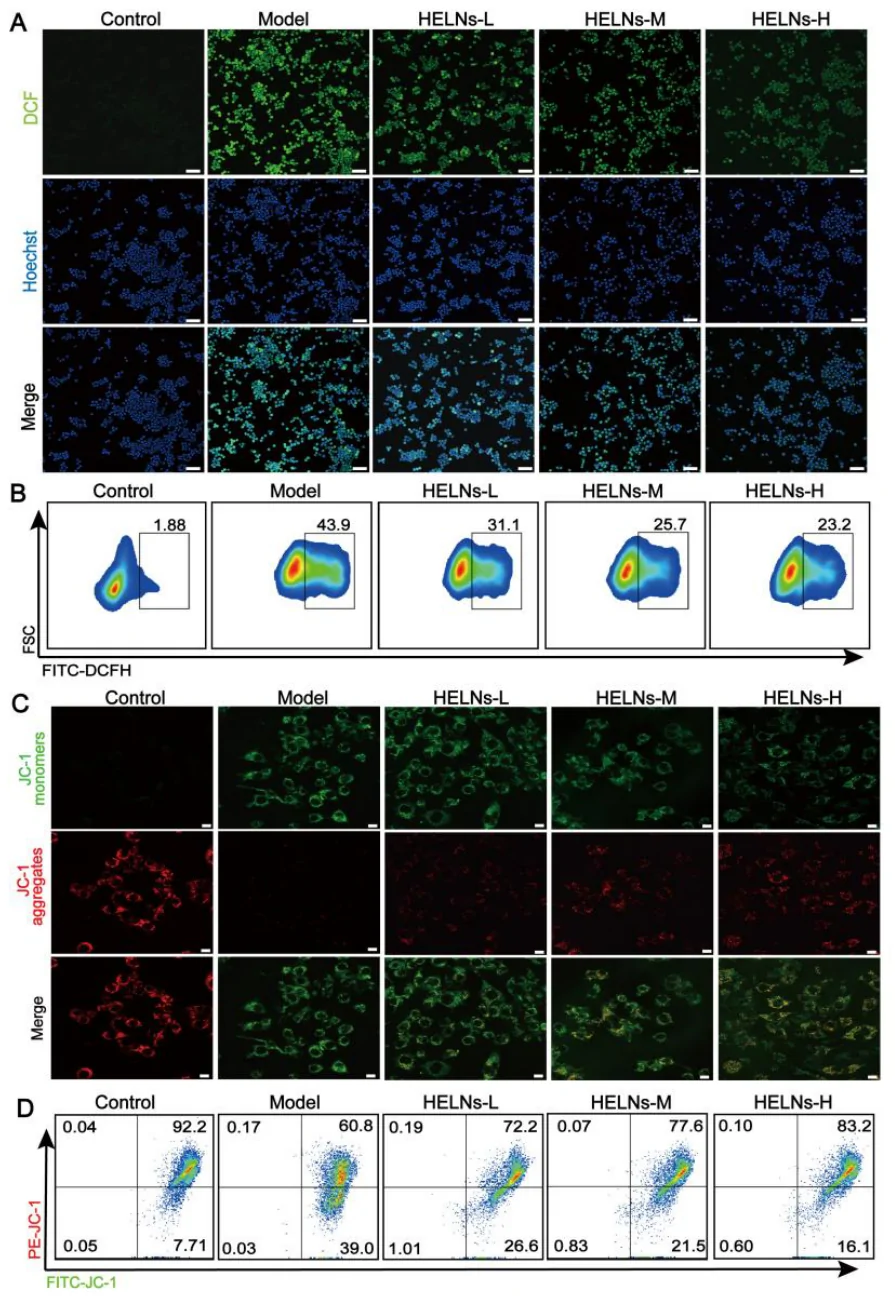
Characteristics of HELNs in scavenging reactive oxygen species and restoring the mitochondrial membrane potential. (**A**) Representative confocal microscopy images showing intracellular ROS levels in different treatment groups via DCFH-DA staining. Scale bar: 50 μm. (**B**) Quantitative analysis of the intracellular ROS fluorescence intensity across different treatment groups. (**C**) Representative JC-1 staining images illustrating the mitochondrial membrane potential in macrophages under different treatments. Scale bar: 10 μm. (**D**) Flow cytometric analysis of JC-1 fluorescence in macrophages following different treatments, *n* = 3.

Oxidative stress is key to activating pro-inflammatory signaling pathways in macrophages, and the accumulation of excessive ROS further exacerbates damage to the inflammatory microenvironment [27,28]. After establishing an oxidative stress model in RAW264.7 cells with 100 ng/mL LPS and 24 h co-incubation with various HELN concentrations, the intracellular ROS levels were detected by DCFH-DA, which revealed that HELNs scavenged ROS in a concentration dependent manner. Confocal microscopy images (Figure 3A) clearly revealed that green fluorescence was significantly enhanced in cells from the LPS-induced model group. Upon the administration of various concentrations of HELNs, the green fluorescence intensity decreased in a dose-dependent manner. The therapeutic effect of HELNs was further illustrated by the semiquantitative fluorescence imaging results (Figure S6A), indicating that HELNs can effectively mitigate the excessive ROS generated by LPS. Flow cytometry analysis (Figure 3B) and semiquantitative flow cytometry results (Figure S6B) confirmed that the dichlorofluorescein (DCF) fluorescence signal in HELNs-H-treated cells was significantly weaker than that in LPS-treated model cells. These results indicated that HELNs significantly reduced LPS-induced ROS levels compared with the LPS model group under the current experimental conditions, suggesting ROS scavenging activity.

Additionally, LPS-induced oxidative stress may result in mitochondrial dysfunction, leading to reduced ATP production and a surge in ROS, which in turn amplifies inflammatory signals, thereby creating a self-perpetuating vicious cycle [29,30]. Therefore, JC-1 fluorescent probe staining was employed to assess the influence of HELNs on the MMP of macrophages under LPS stimulation. LPS-induced JC-1 aggregation (red, high potential) to monomer (green, low potential) conversion in macrophages (Figure 3C), revealing a marked reduction in the MMP. HELNs dose-dependently reversed this effect, shifting JC-1 back toward the aggregate form and thus restoring the membrane potential. Complementary flow cytometry analysis (Figure 3D) corroborated these findings, further demonstrating the ability of HELNs to restore the MMP of LPS-compromised macrophages and enhance their functional capacity. These results indicate that HELNs not only directly scavenge ROS but also suppress oxidative stress at their source by protecting the MMP and preventing the release of mitochondria-derived ROS.

### 3.4. Mechanism of Action of HELNs in the LPS-Induced RAW264.7 Inflammatory Cell Model

**Figure 4:**
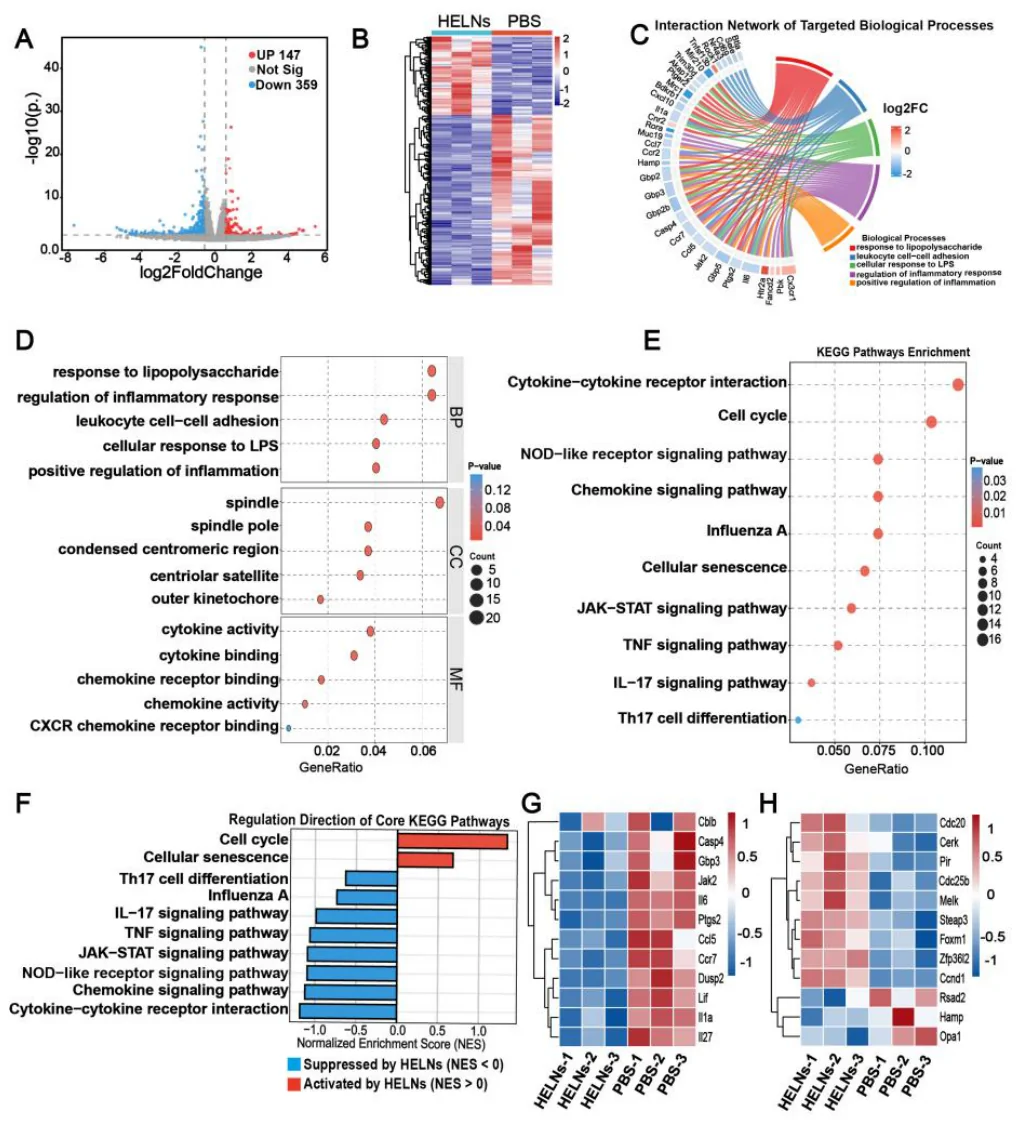
Transcriptomic analysis of inflammatory RAW264.7 cells subjected to HELNs treatment. (**A**) Volcano plot of differentially expressed mRNAs, with the selection criteria for differentially expressed genes (DEGs) being ∣log2(FC)∣ > 0.5 and FDR < 0.05. (**B**) Heatmap showing the expression profiles of all DEGs between the PBS and HELNs treatment groups. (**C**) Chord diagram illustrating gene-pathway associations linked to specific biological processes. (**D**) Gene Ontology (GO) enrichment analysis of the DEGs. (**E**,**F**) The KEGG enrichment analysis and regulatory trend results. (**G**) Inflammation related gene expression heatmap comparing the PBS and HELNs treatment groups. (**H**) Heatmap showing illustrates the expression of genes related to oxidative stress and mitochondria in both the PBS and HELNs treatment groups.

To elucidate the mechanism by which HELNs influence RAW264.7 cells, RNA sequencing (RNA-seq) was performed on cells from both the LPS + PBS and the LPS + HELNs groups. Principal component analysis (PCA) demonstrated high data reproducibility between the two groups (Figure S7A). RNA-seq data from the LPS + PBS and LPS + HELNs groups were analyzed to investigate the molecular changes induced by HELNs treatment. The expressed gene profiles detected in each group were compared using a Venn diagram to illustrate shared and uniquely detected genes (Figure S7B). Differential expression analysis was subsequently performed based on the RNA-seq expression data, and genes with |log_2_(fold change)| > 0.585 and adjusted *p* value (FDR) < 0.05 were considered significantly differentially expressed genes. A total of 147 upregulated genes and 359 downregulated genes were identified and visualized by heatmap and volcano plot analyses (Figure 4A,B). Gene Ontology (GO) enrichment analysis indicated that the significantly altered genes in the HELNs treatment group were predominantly associated with the immune response, cellular processes, and biological regulation (Figure 4C,D). Subsequent Kyoto Encyclopedia of Genes and Genomes (KEGG) enrichment analysis identified several significantly impacted immune-related signaling pathways. Significant downregulation of pro-inflammatory pathways (e.g., TNF and NOD-like receptor signaling) was observed (Figure 4E,F), indicating that HELNs exert anti-inflammatory effects via inhibition of these cascades. Given the strong association between these pathways and inflammatory responses, a heatmap analysis was performed to assess changes in inflammation-related gene expression. As shown in Figure 4G, HELNs reduced the expression of interleukin-6 (IL-6) cytokine family members (IL-6, IL-27, and LIF) as well as the chemokine Ccl5, demonstrating that HELNs effectively suppressed the inflammatory response in RAW264.7 cells. Furthermore, HELNs significantly modulated genes related to mitochondrial function (Figure 4H). The expression levels of Foxm1 and Cdc20, which are essential for regulating the MMP, were notably restored following HELNs treatment [31,32]. Additionally, HELNs upregulated the expression of Opa1 and Melk, contributing to the reduction in excessive intracellular ROS [33,34]. Together, RNA-seq suggested that HELNs may modulate inflammatory and mitochondrial pathways, providing mechanistic clues for future investigation.

### 3.5. In Vivo Biodistribution and Pulmonary Retention of HELNs

**Figure 5:**
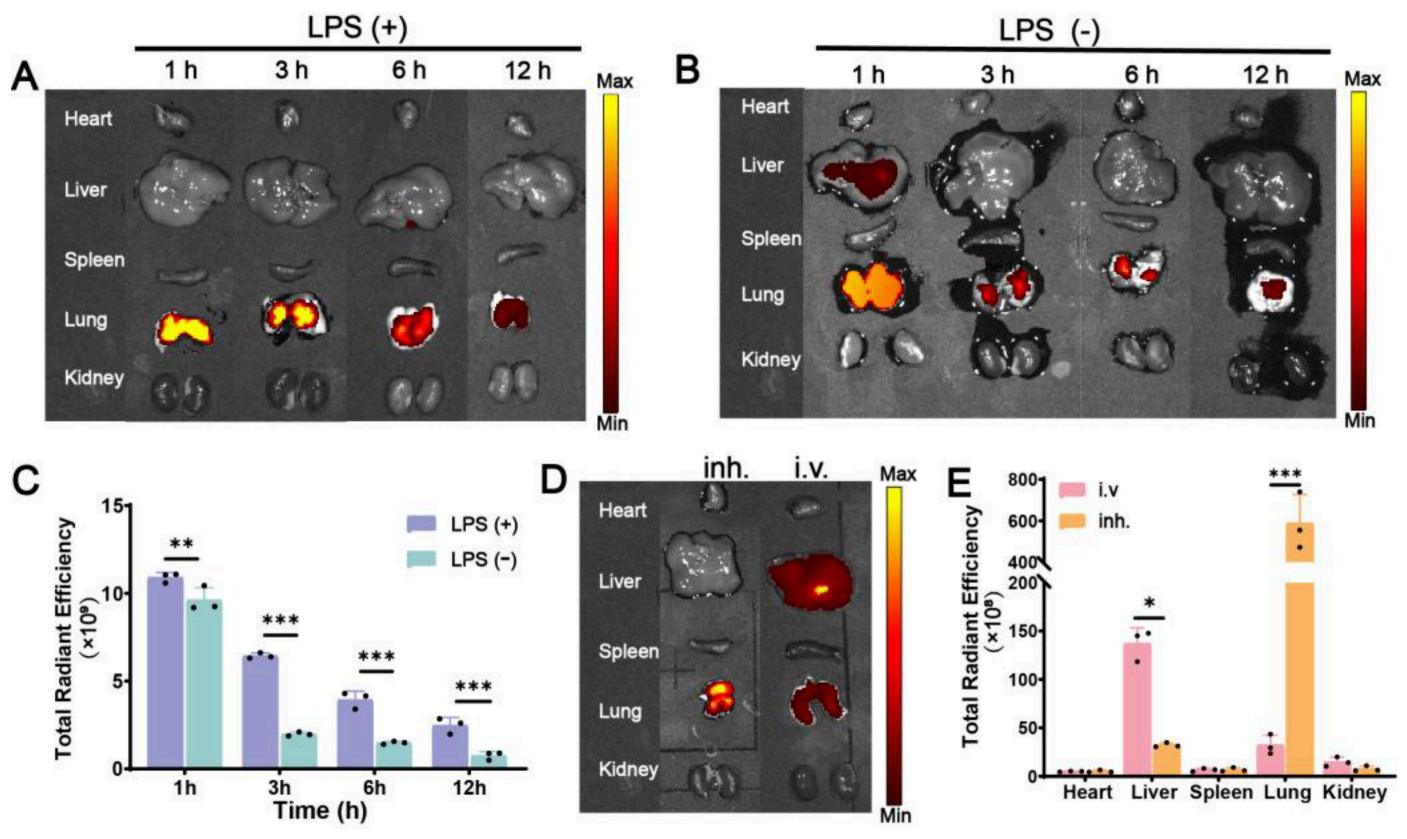
Biodistribution and pulmonary retention of HELNs in vivo. (**A**) Biodistribution of Cy5.5-labeled HELNs in major organs at different time points in LPS-induced acute pneumonia mice. (**B**) Biodistribution of Cy5.5-labeled HELNs in major organs at corresponding time points in mice without LPS treatment. (**C**) Quantitative analysis of the total fluorescence intensity of Cy5.5-labeled HELNs retained in the lungs over time, derived from (**A**,**B**). (**D**) Ex vivo IVIS imaging of Cy5.5-labeled HELNs in various organs at 3 h post-administration via intravenous (i.v.) or inhalation (inh.) administration. (**E**) Quantification of the total fluorescence intensity of Cy5.5-labeled HELNs in various organs 3 h post-administration via i.v. or inh. routes (*n* = 3). Statistical significance: * *p* < 0.05, ** *p* < 0.01, *** *p* < 0.001; *n* = 3.

To evaluate the biodistribution and pulmonary retention of HELNs in vivo, Cy5.5-labeled HELNs were synthesized and administered via inhalation 12 h following either LPS or control (non-LPS) stimulation [35,36]. Heart, liver, spleen, lung, and kidney tissues were collected at serial time points after inhalation for in vivo imaging analysis. The findings revealed persistent and significantly elevated fluorescence signals in the lungs of mice with acute lung injury at multiple time points, compared with those in the lungs of control (non-LPS) mice (Figure 5A,B). Quantitative analysis of the time-dependent total fluorescence intensity of Cy5.5 labeled HELNs retained in the lungs revealed a significant increase in HELN accumulation within inflamed lung tissue (Figure 5C), which may be attributed to alterations in the pulmonary inflammatory microenvironment. To further elucidate the enhanced lung-specific distribution achieved through nebulized delivery, a comparative biodistribution analysis was performed following both intravenous injection and inhalation. The results indicated that intravenous administration led to predominant accumulation in the liver, with significantly lower enrichment in the lungs than did inhalation (Figure 5D,E). The fluorescence intensity observed in the lungs of the inhalation group was substantially higher than that of the intravenous group, demonstrating that nebulized administration significantly enhances pulmonary bioavailability. Thus, noninvasive inhalation of therapeutic nanoparticles represents a promising approach for treating pulmonary diseases. This method facilitates direct delivery to the lung tissue, enables better local enrichment, and minimizes systemic distribution, thereby improving therapeutic precision and efficacy while reducing potential side effects.

Following the assessment of the biodistribution and pulmonary retention of HELNs, a comprehensive assessment of their potential in vivo toxicity was conducted. Healthy mice received the formulation via inhalation, and 24 h after the completion of administration, histopathological, hematological, and serum biochemical evaluations were performed. No inflammatory infiltration, necrosis, fibrosis, or drug related pathological changes were observed in H&E stained sections of heart, liver, spleen, lung, and kidney tissues (Figure S8A). Compared with the control group, the HELNs treated group presented normal RBC, WBC, and HGB levels, with no signs of anemia or hematological toxicity (Figure S8B–D). Notably, the WBC count in the HELNs group remained within the normal range, suggesting no impairment of immune function. In contrast, the WBC count was markedly decreased in the dexamethasone (Dex) treated positive control group (Figure S8C). Additionally, we measured serum biomarkers for liver (AST, ALT, TBIL) and kidney (CREA, BUN) function (Figure S8E–I). No statistically significant differences in these parameters were observed across groups, indicating that inhaled HELNs do not cause significant liver or kidney damage, with normal TBIL levels further ruling out bile excretion disorders or severe hepatocellular injury. Overall, these findings demonstrate that HELNs have a favorable safety profile in healthy mice, with no notable histopathological, hematological, or hepatorenal toxicity, thereby providing a robust experimental basis for their further application.

### 3.6. Therapeutic Study of LPS-Induced Acute Lung Injury (ALI)

**Figure 6:**
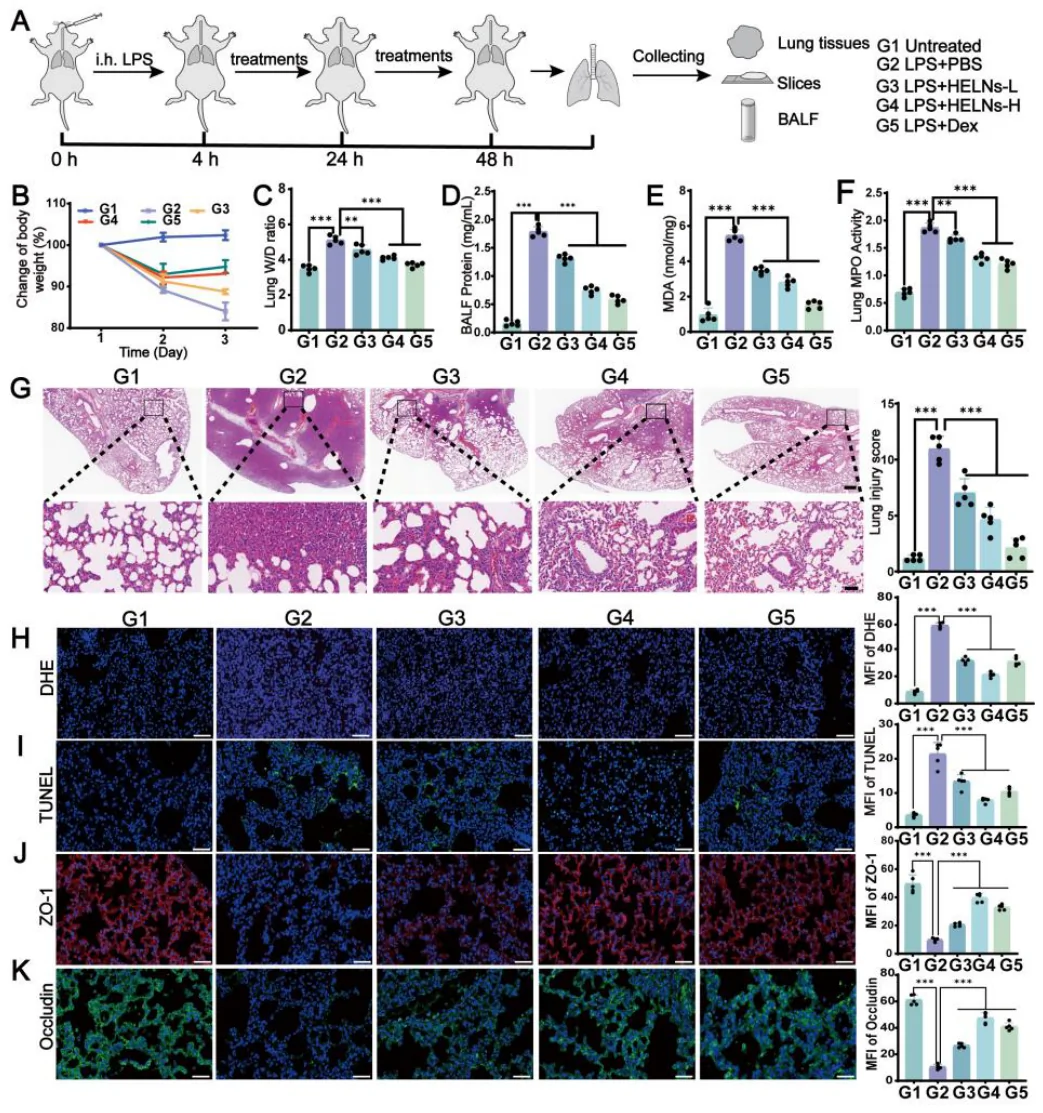
Therapeutic effects of HELNs on LPS-induced ALI. (**A**) Schematic illustration of the animal model and treatment protocol. Male BALB/c mice were randomly divided into five groups: untreated control group (G1), LPS-induced disease model control group (20 μL,5 mg/kg) (G2), LPS + low-dose HELNs group (0.5 mg/mouse) (G3), LPS + high-dose HELNs group (1 mg/mouse) (G4), and LPS + Dex group (0.4 mg/mouse) (G5). (**B**) Body weight changes in the mice over the treatment period. (**C**) Lung wet weight to dry weight ratio. (**D**) The protein concentration in the BALF. (**E**) The concentration of malondialdehyde (MDA) in lung tissue. (**F**) Myeloperoxidase (MPO) levels in lung homogenates. (**G**) Hematoxylin and eosin (H&E) staining analysis of lung tissues post-treatment, with a wide-field view (scale bar: 0.5 mm) and magnified regions (scale bar: 50 μm). (**H**) Representative DHE (ROS, red) and DAPI (nuclei, blue) staining of lung tissue. Scale bar: 50 μm. (**I**) Representative TUNEL staining images and quantitative mean fluorescence intensity (MFI). Scale bar: 50 μm. (**J**) Immunofluorescence staining of ZO-1 (red) and the corresponding quantitative MFI. Scale bar: 50 μm. (**K**) Immunofluorescence staining of occludin (green) and the quantitative MFI are displayed. Scale bar: 50 μm. Statistical significance: ** *p* < 0.01, *** *p* < 0.001; *n* = 5.

These favorable in vitro results prompted us to explore the therapeutic potential of HELNs in LPS-induced ALI mice. Specific-pathogen-free (SPF) male BALB/c mice were randomly divided into five groups in this experiment: normal control (untreated) (G1), LPS-induced disease model control (model) (G2), LPS + low-dose HELNs (HELNs-L) (0.5 mg/mouse) (G3), LPS + high-dose HELNs (1 mg/mouse) (HELNs-H) (G4), and LPS + dexamethasone (0.4 mg/mouse) (Dex) (G5). LPS was instilled intratracheally at a dose of 5 mg/kg (20 μL/mouse) to induce ALI in mice. This was followed by two rounds of nebulization with a custom-built device (Figure S9). After 24 h, pulmonary function indices were measured and tissue samples were collected for analysis (Figure 6A). As shown in Figure 6B, during the treatment period, HELNs-L, HELNs-H, and Dex alleviated body weight loss in BALB/c mice to varying degrees, suggesting that HELNs and Dex improved the overall physiological status of the mice. A multidimensional lung analysis was conducted to confirm the therapeutic efficacy of the drugs against ALI. Persistent inflammation raises alveolar-capillary barrier permeability, causing protein-rich edema fluid to accumulate in alveolar spaces. Therefore, we assessed pulmonary edema and vascular permeability changes by measuring the BALF protein concentration and the lung wet/dry (W/D) weight ratio. LPS significantly increased vascular permeability and induced severe pulmonary edema, as reflected by an elevated W/D ratio and increased BALF protein content (Figure 6C,D). Treatment with different concentrations of HELNs and Dex alleviated pulmonary edema to varying degrees, with the HELNs-H and Dex groups showing more pronounced effects, suggesting that HELNs may protect the alveolar–capillary barrier through their antioxidant capacity.

Persistent inflammation and oxidative stress often form a vicious cycle during disease progression [37]. To investigate whether the treatment interrupted this cycle, we measured the levels of myeloperoxidase (MPO) and malondialdehyde (MDA). After LPS challenge, both MPO and MDA levels were significantly elevated, indicating oxidative damage in the lungs (Figure 6E,F). HELNs-L treatment slightly reduced MPO and MDA levels, whereas HELNs-H and Dex treatment led to a substantial decrease in both, highlighting the remarkable ability of HELNs to alleviate oxidative stress. To visually assess the therapeutic effects on the lungs, we performed H&E staining to evaluate lung tissue injury and used a standardized scoring system to quantify the severity of injury. The LPS-induced disease model control group exhibited severe lung pathology, including extensive inflammatory infiltration, alveolar wall thickening and collapse, along with interstitial congestion and edema. Both the HELNs and Dex treatment groups demonstrated partial attenuation of inflammatory infiltration and alleviation of interstitial edema. To further substantiate these findings, we employed the fluorescent probe dihydroethidium (DHE) to assess the capacity for scavenging ROS. As depicted in Figure 6H, the DHE fluorescence signal was significantly intensified in the model group, indicating excessive ROS production in the ALI model. Conversely, the DHE fluorescence intensity was markedly diminished in both the HELNs-H and Dex groups. Semiquantitative analysis further indicated that HELNs-H exhibited superior efficacy to Dex, highlighting the enhanced ROS scavenging ability of HELNs. Notably, the Dex group presented largely intact alveolar structures, minimal inflammatory cell infiltration, and only mild alveolar septal thickening (Figure 6G), with the lowest lung injury score. The HELNs-H group displayed only focal alveolar wall thickening with mild hemorrhage and inflammatory infiltration. These results indicate that HELNs can block the vicious cycle of inflammation and oxidative stress by inhibiting pro-inflammatory mediators, reducing ROS generation, and enhancing cellular tolerance to oxidative stress, thereby effectively alleviating pulmonary edema and promoting tissue repair.

On the basis of the above findings, we further explored the impact of ROS on the alveolar–capillary barrier. In ALI, accumulated ROS and inflammation promote epithelial/endothelial cell apoptosis, which degrades tight junction proteins (ZO-1, occludin) and disrupts the alveolar–capillary barrier [38,39,40]. To further elucidate the mechanism by which HELNs alleviate pulmonary edema, we assessed cell apoptosis and tight junction protein integrity. The model group exhibited significantly stronger TUNEL fluorescence in lung sections (Figure 6I), indicating more apoptotic cells. In contrast, the HELNs-H and Dex groups exhibited markedly reduced apoptosis, demonstrating strong anti-apoptotic effects. The results of the immunofluorescence staining (Figure 6J,K) revealed that in the control group, ZO-1 displayed high fluorescence intensity with a uniform linear distribution along the cell margins. After the LPS stimulation, fluorescence intensity was significantly reduced, and a discontinuous staining pattern was observed. HELNs and Dex treatment improved ZO-1 fluorescence intensity and partially restored cell membrane staining, with the HELNs-H group showing the most pronounced improvement in both staining intensity and continuity. Similar trends were observed for occludin expression. These results indicate that HELNs and Dex can effectively restore the alveolar–capillary barrier.

### 3.7. Immune Response and Assessment

**Figure 7:**
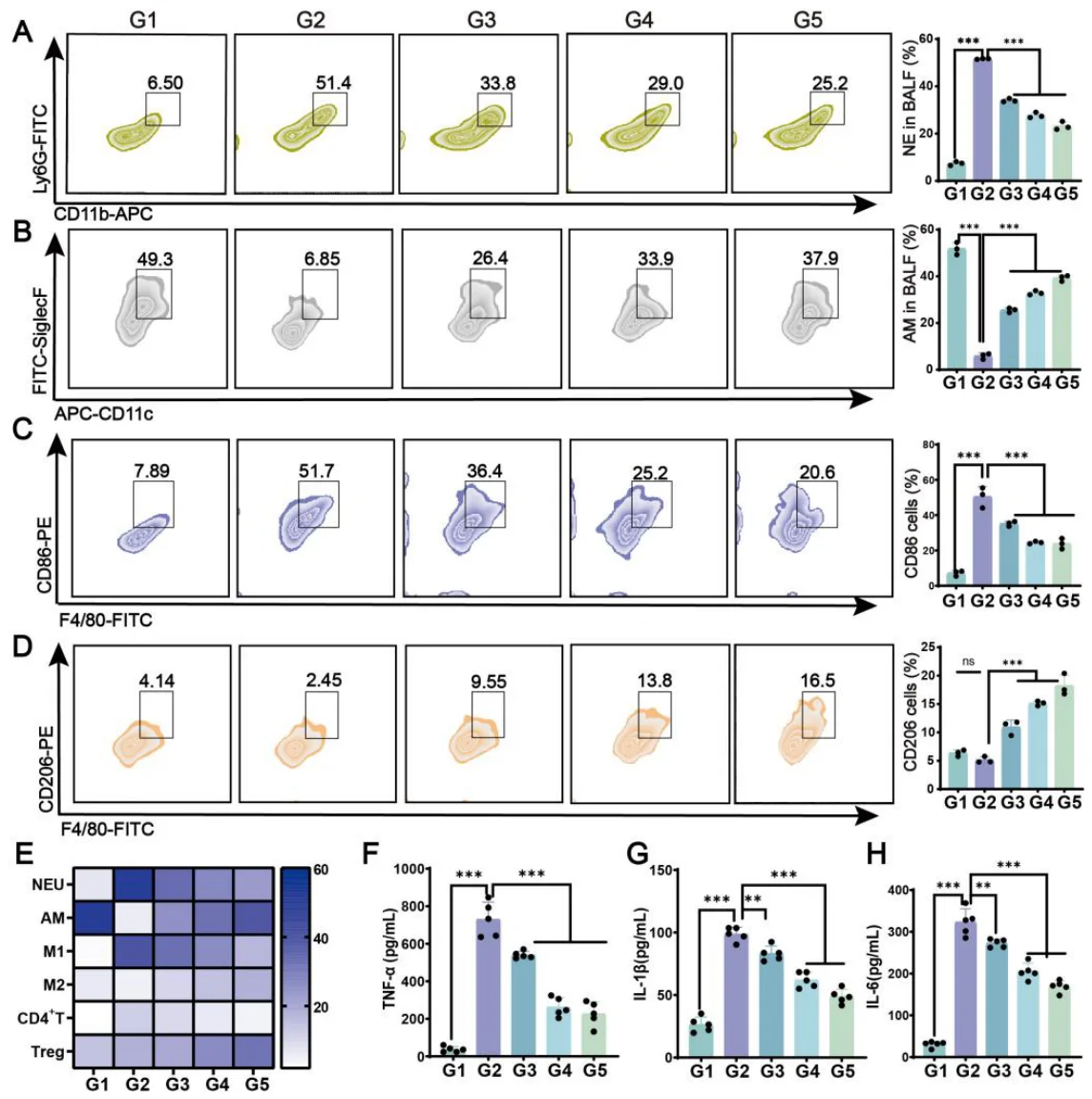
Immune response and assessment. (**A**) Flow cytometry plots and quantified percentages of neutrophils (NE) in the BALF. (**B**) Flow cytometry plots and quantified percentages of alveolar macrophages (AMs). (**C**) Flow cytometry plots and quantified percentages of M1 macrophages in lung tissue. (**D**) Flow cytometry plots and quantified percentages of M2 macrophages in lung tissue. (**E**) Heatmap illustrating the quantified percentages of cells within the innate and adaptive immune systems. (**F**–**H**) Concentrations of the cytokines TNF-α, IL-1β, and IL-6 in the BALF. Statistical significance: ** *p* < 0.01, *** *p* < 0.001, ns means no significant; *n* = 5.

To determine the observed therapeutic benefits, we analyzed the immune response by categorizing it into innate and adaptive immunity and evaluating both components. The primary elements of the innate immune system, macrophages and neutrophils, play pivotal roles as regulators of inflammation and tissue damage [41,42]. Using flow cytometry, we assessed the proportions of neutrophils (NE) and alveolar macrophages (AM) in the BALF (Figure 7A,B; Figures S10 and S11). Consistent with expectations, the model group had heavy inflammatory cell infiltration, with the percentage of Ly6G^+^CD11b^+^ cells among total BALF cells increasing to approximately 51.4% and that of alveolar macrophages (CD11c^+^ Siglec-F^+^) decreasing to 6.85%. In the treatment cohorts, the HELNs-H group had a reduction in the neutrophil proportion to 29.0%, whereas the positive control Dex group had a further reduction to 25.2%. Concurrently, the alveolar macrophage proportion increased to 33.9% in the HELNs-H group and to 37.9% in the Dex group. These findings demonstrate that HELNs exert a favorable immunomodulatory effect on the innate immune system, suggesting that HELNs-H exhibit efficacy comparable to that of Dex. As shown in Figure 7C,D and Figures S12 and S13, lung tissue from HELNs-H-treated mice had a higher percentage of M2 macrophages and a lower percentage of M1 macrophages. These findings suggest that HELNs reduce neutrophil infiltration and promote M2 polarization, contributing to the modulation of the inflammatory microenvironment.

We evaluated the impact of HELNs on adaptive immune responses by using flow cytometry to analyze various T-cell subsets, including CD8^+^ T cells, CD4^+^ T cells, and regulatory T cells (Tregs). Previous studies have indicated that overactivation of CD8^+^ T cells can exacerbate pulmonary injury [43]. Our findings demonstrated that LPS administration increased the recruitment of CD8^+^T cells (CD3^+^ CD8^+^) within lung tissue, whereas HELNs treatment reduced the proportion of these cells. Furthermore, overactivation of effector CD4^+^ T cells is associated with irreversible tissue damage, while Tregs play a crucial role in modulating excessive immune responses and facilitating tissue repair [44]. As illustrated in Figures S14 and S16A,B, the model group displayed a significantly elevated count of CD4^+^ T cells, indicating an overactive adaptive immune response induced by LPS. In the HELNs-treated group, the percentage of CD4^+^ T cells was reduced, whereas the Treg level was increased (Figures S15 and S16C,D). Notably, HELNs treatment effectively suppressed the increase in CD4^+^ T cells and elevated Treg levels. This study demonstrated that HELNs exert an immunomodulatory effect by potentially mitigating excessive inflammatory responses or autoimmune damage through the restoration of CD4^+^ T-cell subset equilibrium, particularly via the enhancement of Treg-mediated immunosuppressive functions. The heatmap in Figure 7E illustrates alterations in immune cell populations as determined by flow cytometry. Additionally, the expression of pro-inflammatory cytokines, including TNF-α, IL-1β, and IL-6, in the BALF was significantly reduced in the HELNs-treated groups (Figure 7F–H). HELNs effectively modulate both innate and adaptive immune responses by diminishing neutrophil infiltration, inhibiting CD4^+^/CD8^+^ T-cell accumulation, promoting M2 macrophage polarization, and stimulating Treg expansion, which in turn creates an immune microenvironment conducive to inflammation resolution and reduces the levels of pro-inflammatory cytokines (TNF-α, IL-1β, and IL-6).

## 4. Discussion

Here, we report that HELNs—PENs obtained from the medicinal and edible plant *Houttuynia cordata*—can be administered via noninvasive nebulization. This approach significantly ameliorates acute lung injury caused by LPS, as demonstrated for the first time. The HELN formulation displayed favorable aerodynamic properties for pulmonary delivery, though the partial particle aggregation observed during nebulization highlights an opportunity for future optimization of formulation stability or nebulization parameters. Compared with intravenous administration, nebulized delivery achieved significantly higher pulmonary fluorescence retention, supporting the superior lung delivery efficiency of the inhalation route. Given that the enhanced pulmonary signal in inflamed lungs may partly reflect increased vascular permeability in the injured tissue, future studies using more specific labeling or pharmacokinetic approaches will be helpful to further differentiate intact nanoparticle deposition from passive extravasation. HELNs were efficiently taken up by macrophages in vitro, where they exert dual protective effects—scavenging excessive ROS [4] and restoring the MMP—while simultaneously modulating macrophage polarization from the M1 phenotype to the M2 phenotype [9]. In the murine ALI model, nebulized HELNs significantly alleviated pulmonary edema, mitigated oxidative tissue damage, restored alveolar-capillary barrier integrity, and comprehensively rebalanced pulmonary immune homeostasis, with no significant systemic or organ toxicity observed in vivo. Collectively, these findings establish nebulized HELNs as a safe, effective, and highly scalable therapeutic strategy for ALI.

Several important limitations of this study must be acknowledged. First, the key bioactive constituents responsible for the observed antioxidant and immunomodulatory activities of HELNs remain unidentified. Although metabolomic profiling revealed that HELNs are enriched with lipids, organic acids, and diverse bioactive metabolites inherited from the parental plant, the specific molecular entities driving ROS scavenging, mitochondrial protection, and macrophage reprogramming have not been isolated or functionally validated. Therefore, activity-guided fractionation combined with targeted metabolomics and lipidomics will be essential to pinpoint the pharmacologically active components and define their relative contributions to overall therapeutic efficacy. Second, the molecular mechanisms underlying the therapeutic effects were inferred primarily from global transcriptomic analyses, which highlighted the downregulation of pro-inflammatory TNF signaling and NOD-like receptor signaling pathways [6] and the restoration of mitochondrial function-related gene expression. However, these transcriptomic findings should be viewed as hypothesis-generating, and future work will need to confirm the causal links between the identified pathway alterations and the anti-inflammatory phenotypes through targeted genetic or pharmacological intervention, as well as validate key candidate genes at the mRNA and protein levels. Further rigorous mechanistic studies are warranted to elucidate the precise signaling networks engaged by HELNs. Third, the in vivo therapeutic efficacy was evaluated exclusively in the LPS-induced murine ALI model. While this model is the most widely used preclinical platform for acute pulmonary inflammation research [3], it cannot fully recapitulate the pathophysiological complexity of clinical ALI/ARDS, which arises from heterogeneous etiologies including bacterial pneumonia, sepsis, and trauma. Validation of HELNs’ efficacy in additional clinically relevant models—such as live bacterial pneumonia, cecal ligation and puncture (CLP)-induced sepsis [27], and large-animal systems—will be critical to strengthen the translational relevance of our findings. Fourth, although in vitro assays demonstrated efficient internalization of HELNs by macrophages, direct evidence for their uptake by pulmonary macrophages in vivo remains to be established; current conclusions regarding in vivo macrophage modulation are therefore based on indirect phenotypic readouts, and future studies employing fluorescence co-localization or flow cytometric analysis of pulmonary macrophages will be important to validate this mechanism directly. Fifth, because the present proof-of-concept study relied on a single large-scale extraction batch, inter-batch variability in particle-to-protein ratio and other quality attributes could not be assessed. Future studies employing multiple independently prepared batches will therefore be necessary to establish batch-to-batch consistency and to define appropriate quality control criteria for translational development. Finally, this study remains at the preclinical proof-of-concept stage, and comprehensive pharmacokinetic, pharmacodynamic, and long-term safety evaluations—together with strategies to mitigate nebulization-induced particle aggregation—are required before clinical translation can be considered.

The findings from this work also point toward several high-priority future research directions. Given their intrinsic therapeutic bioactivity and nanoscale vesicle architecture, HELNs represent an ideal versatile platform for drug delivery. Encapsulating anti-inflammatory agents (e.g., dexamethasone), antibiotics, or other hydrophobic therapeutics within HELNs could yield synergistic therapeutic effects and further improve ALI/ARDS treatment outcomes while minimizing systemic side effects associated with high-dose systemic drug administration [45]. Furthermore, the surface of HELNs can be functionalized with delivery ligands—such as cyclic RGD peptides that specifically bind integrin αvβ3 overexpressed on activated macrophages and inflamed endothelium [46]—to enhance site-specific delivery to inflamed pulmonary tissues. The dual redox-modulating and immunomodulatory mechanisms demonstrated by HELNs also suggest broader therapeutic potential in other pulmonary disorders driven by oxidative stress and dysregulated inflammation, including chronic obstructive pulmonary disease (COPD), asthma, and idiopathic pulmonary fibrosis. Finally, to facilitate clinical translation, future efforts should focus on establishing standardized, good manufacturing practice (GMP)-compliant manufacturing protocols; defining critical quality attributes (e.g., particle size distribution, zeta potential, protein/metabolite composition, and biological potency assays); conducting comprehensive preclinical safety and efficacy evaluations in large-animal models; and optimizing nebulization conditions or formulation parameters to address particle aggregation during aerosolization.

## 5. Conclusions

In this study, exosome-like nanoparticles derived from medicinal and edible plant HELNs were successfully isolated, characterized, and delivered via noninvasive nebulization for the treatment of ALI. HELNs exhibit a characteristic bilayer membrane vesicle structure, appropriate particle size distribution, favorable low-temperature storage stability, and excellent biocompatibility. When nebulized, HELNs efficiently accumulate in inflamed lung tissue and are expected to be taken up by macrophages. Mechanistically, HELNs demonstrated synergistic multi-target therapeutic effects. Specifically, HELNs directly scavenge excessive ROS and restore the MMP, thereby interrupting the vicious cycle of oxidative stress and inflammation at its source. Moreover, HELNs repolarized macrophages from M1 to M2; significantly suppressed the infiltration of neutrophils, CD4^+^ T cells, and CD8^+^ T cells in the lungs; and expanded Tregs. Consequently, these effects create a microenvironment conducive to inflammation resolution and reduce the levels of pro-inflammatory cytokines (TNF-α, IL-1β, and IL-6).

Furthermore, HELNs restored barrier integrity by reducing epithelial/endothelial apoptosis and increasing ZO-1/occludin expression, thereby alleviating pulmonary edema and reducing protein leakage. In an LPS-induced mouse model of ALI, nebulized HELNs significantly alleviated lung histopathological damage, reduced oxidative stress markers, and improved the overall physiological condition of the animals. RNA-seq confirmed HELNs suppressed the TNF and NOD-like receptor pathways and modulated mitochondrial genes, explaining their molecular anti-inflammatory/antioxidant effects. In vitro/in vivo safety tests showed no hemolysis, organ toxicity, or immunosuppression, supporting the clinical potential. In conclusion, nebulized PENs derived from *Houttuynia cordata* offer a promising therapeutic strategy for acute lung injury. These nanoparticles synergistically combine antioxidant, anti-inflammatory, and immunomodulatory functions to facilitate alveolar–capillary barrier repair, offering a safe, effective, and scalable option. This approach shows potential for the clinical management of ALI/ARDS and other inflammatory pulmonary diseases.

## Data Availability

All data supporting the findings of this study are available from the corresponding authors upon request.

## Supplementary Materials

The following supporting information can be downloaded at: https://www.mdpi.com/article/10.3390/pharmaceutics18101235/s1.

## Abbreviations

ALI, Acute Lung Injury; ALT, Alanine Aminotransferase; ARDS, Acute Respiratory Distress Syndrome; BALF, Bronchoalveolar Lavage Fluid; BUN, Blood Urea Nitrogen; CCK-8, Cell Counting Kit 8; CREA, Creatinine; Dex, Dexamethasone; DHE, Dihydroethidium; DLS, Dynamic Light Scattering; H&E, Hematoxylin and Eosin; HELNs, *Houttuynia cordata* Exosome-like Nanoparticles; HGB, Hemoglobin; IL-1β, Interleukin-1 beta; IL-6, Interleukin-6; LPS, Lipopolysaccharide; M1, pro-inflammatory Macrophage Phenotype; M2, Anti-inflammatory Macrophage Phenotype; MPO, Myeloperoxidase; MDA, Malondialdehyde; MMP, Mitochondrial Membrane Potential; NTA, Nanoparticle Tracking Analysis; PENs, Plant-derived Exosome-like Nanoparticles; RAW264.7, Mouse Leukemic Monocyte Macrophage Cell Line; RBC, Red Blood Cell; SDS-PAGE, Sodium Dodecyl Sulfate-Polyacrylamide Gel Electrophoresis; ROS, Reactive Oxygen Species; TBIL, Total Bilirubin; TEM, Transmission Electron Microscopy; TNF-α, Tumor Necrosis Factor-alpha; TUNEL, Terminal Deoxynucleotidyl Transferase; dUTP, Nick-End Labeling; WBC, White Blood Cell; ZO-1, Zonula Occludens-1.
