# Supplementary material for: Inhalable Plant Exosome-like Nanoparticles from Houttuynia cordata Ameliorate Acute Lung Injury via Redox Modulation and Immune Reprogramming
Source: Pharmaceutics. 2026 Sep 29;18(10):1235. doi: 10.3390/pharmaceutics18101235 (PMC13708109; doi:10.3390/pharmaceutics18101235)
Supplement: Supplementary file 1 [file pharmaceutics-18-01235-s001.zip › pharmaceutics-4455718-supplementary.pdf]

Supplementary Information

Inhalable Plant Exosome-Like Nanovesicles from Houttuynia cordata Ameliorate Acute Lung Injury via Redox Modulation and Immune Reprogramming

Ting Yin[a,b,#], Junjian Hu[a,b,#], Lijuan Xu[a,b], Shichen Huang[a,c], Bao Zhang[a,b], Long Qiu[a,b], Yanlei Liu[a,b], Daxiang Cui[a,b], Zhengjie Zhou[a,c], Jincheng Zeng[a,c], Menglei Zha[a,d*]

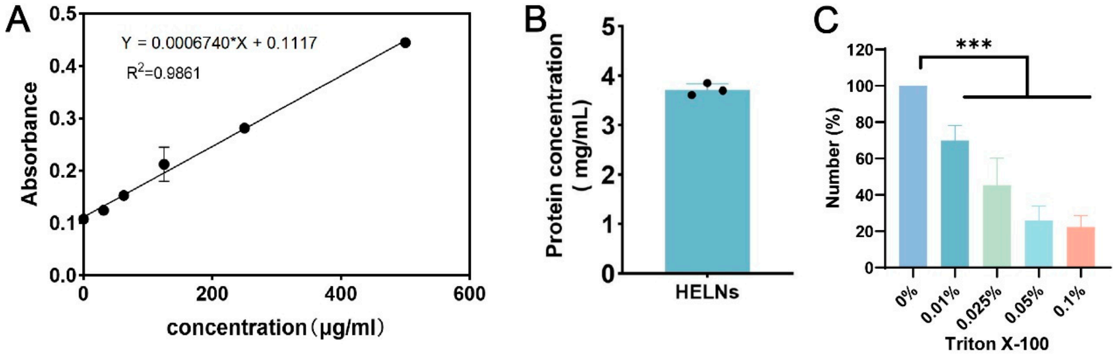

Supporting Information S1. (A) Standard curve graph for quantifying HELNs using the BCA method. (B) Quantifying the protein concentration of HELNs using the BCA method. (C). Triton X-100 detection of lipid membrane structure of HELNs. Data are presented as mean ± SD (ns indicates no statistical significance; *P < 0.05, **P < 0.01, ***P < 0.001; n = 3).

.

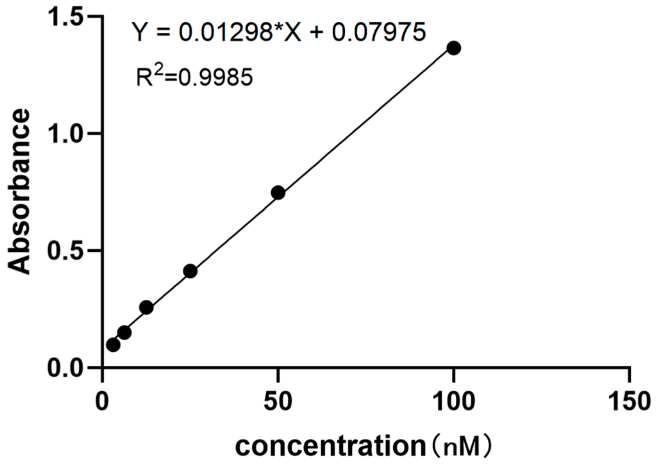

Supporting Information S2. Using a kit to establish a standard curve for NO concentration gradient.

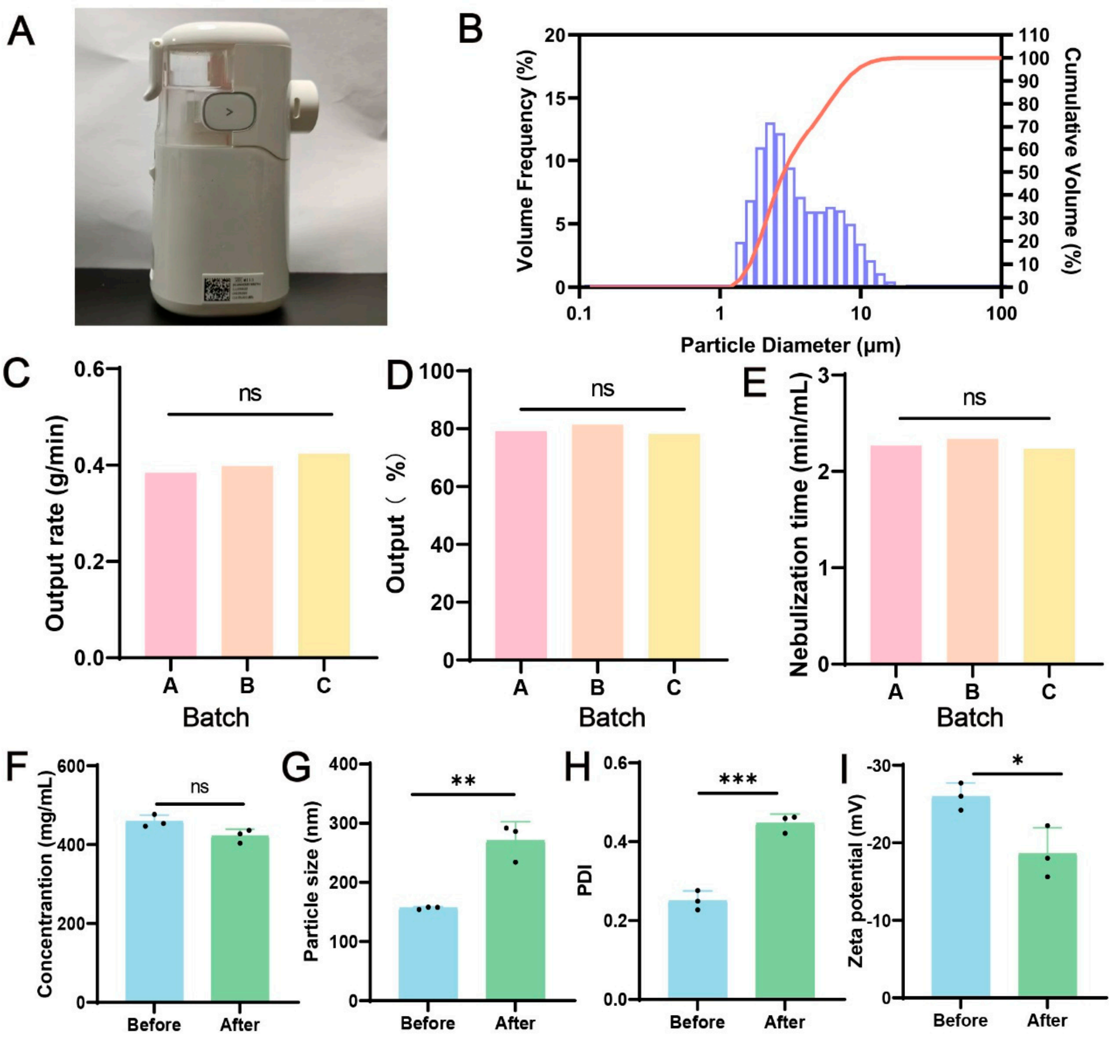

Supporting Information S3. Aerodynamic and aerosolization performance evaluation. (A) Nebulizer for aerodynamic determination of HELNs. (B) The droplet size distribution of nebulized HELNs evaluated by Spraytec laser diffraction analysis. (C) output rate, (D) output

efficiency, and (E) Nebulization time of HELNs in 3 individual batches. (F) protein concentration, (G) Particle size, (H) PDI, and (I) zeta potential of HELNs (Before) and atomized droplets (After). *P < 0.05, **P < 0.01, ***P < 0.001; n = 3).

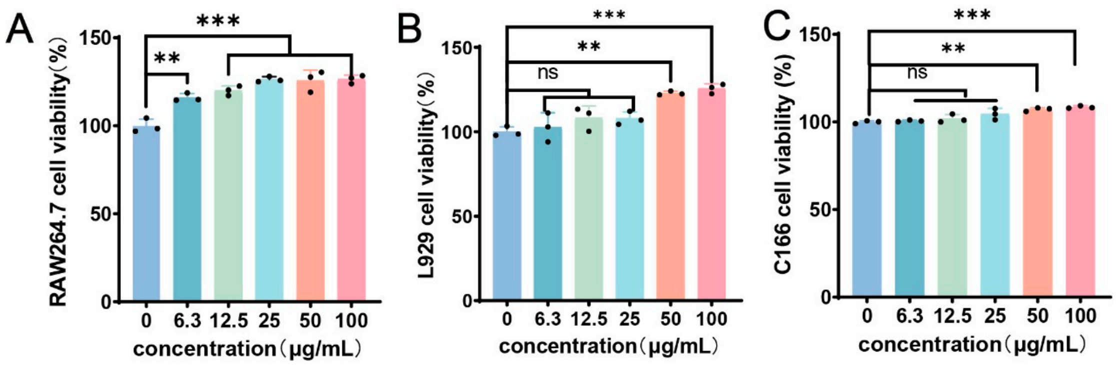

Supporting Information S4. (A) Cytotoxic effect of HELNs on RAW264.7 cells. (B) Cytotoxic effect of HELNs on L929 cells. (C) Cytotoxic effect of HELNs on C166 cells. Data are presented as mean ± SD (ns indicates no statistical significance; *P < 0.05, **P < 0.01, ***P < 0.001; n = 3).

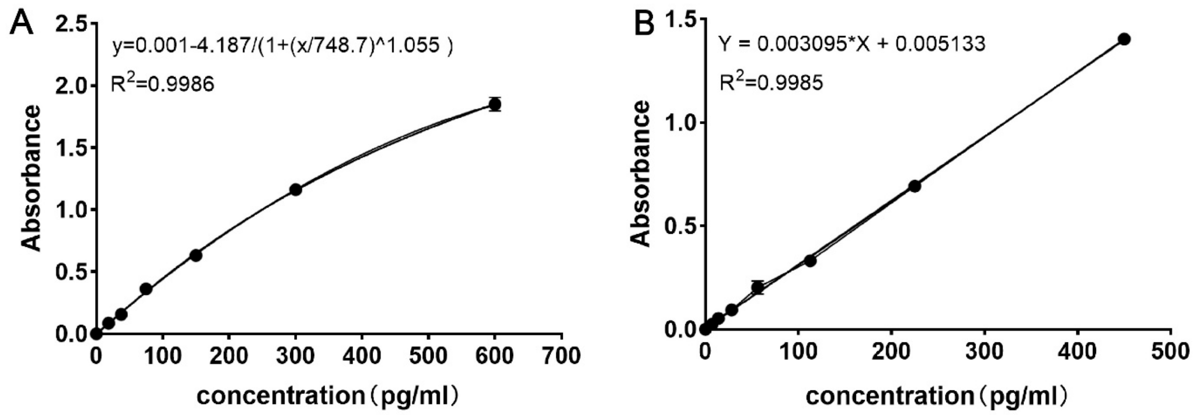

Supporting Information S5. (A) Using an ELISA kit to establish a standard curve graph for TNF-α concentration. (B) Using an ELISA kit to establish a standard curve graph for IL-6 concentration.

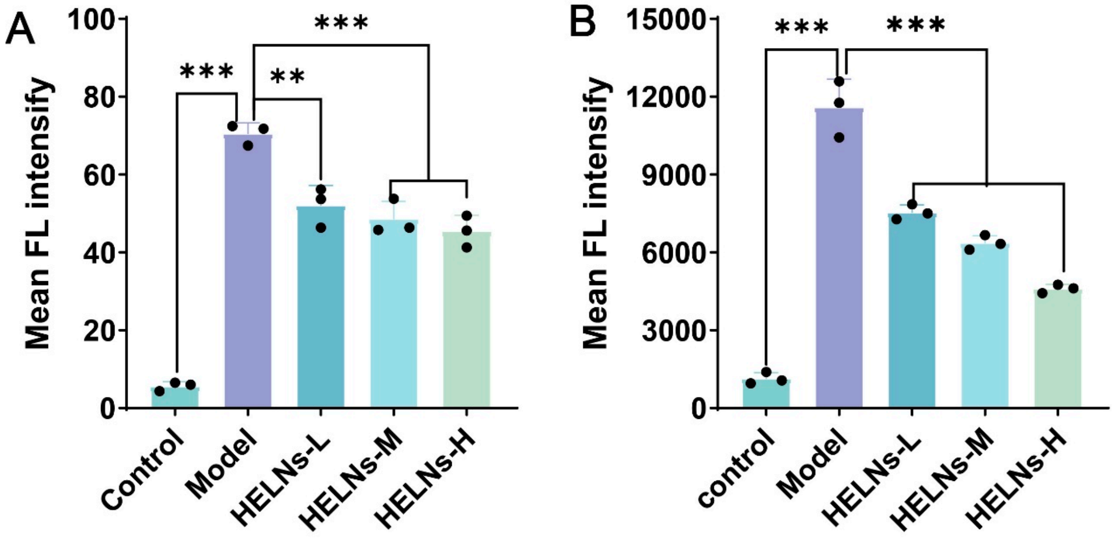

Supporting Information S6. (A) Semi-quantitative result graph of ROS from confocal microscopy. (B) Semi-quantitative result graph of ROS by flow cytometry. Data are presented as mean ± SD (ns indicates no statistical significance; *P < 0.05, **P < 0.01, ***P < 0.001; n = 3).

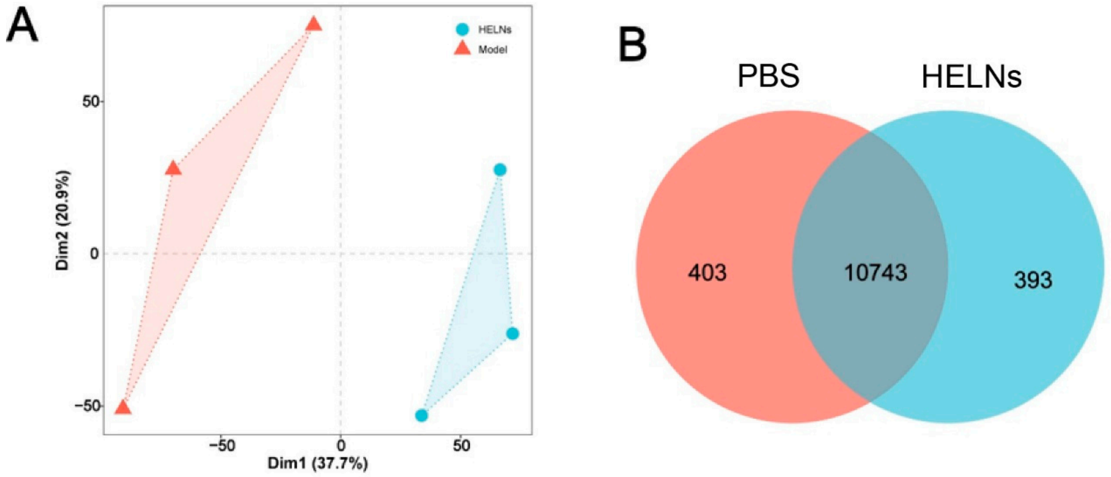

Supporting Information S7. (A) The PCA plot shows the differences between the PBS-treated group and the HELNs-treated group in the ALI model (n=3). (B) The Venn diagram illustrates the overlap and group-specific expressed genes detected between the Model group and the HELNs-treated group based on RNA-seq expression profiles. Genes were considered expressed when the summed raw read counts across the three biological replicates within each group were greater than zero.

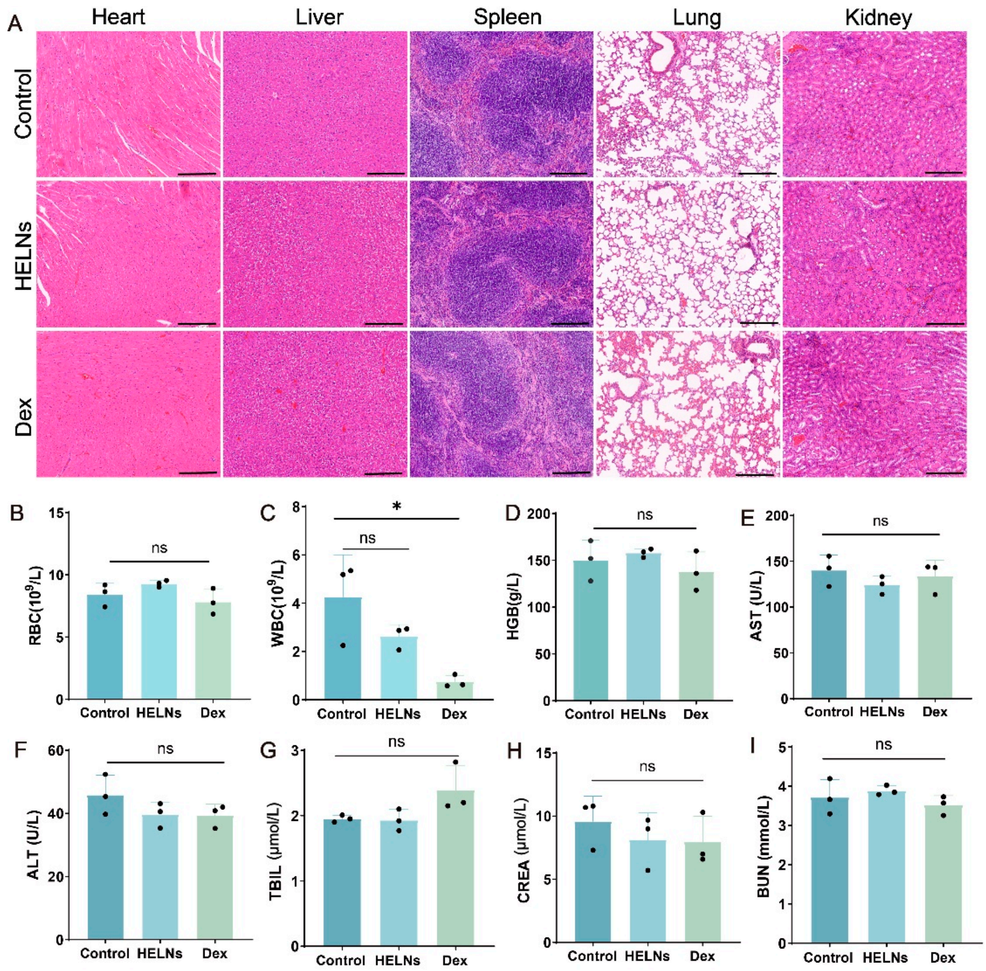

Supporting Information S8. Biosafety Assessment of HELNs. (A) H&E-stained sections of major organs from mice after different treatments. Scale bar: 200 μm. Hematological parameters: (B), (C), (D) represent the concentrations of RBC, WBC, and HGB in mouse blood after different treatments, respectively. Biochemical indicators: (E), (F), (G), (H), (I) represent the serum levels of AST, ALT, TBIL, CREA, and BUN after different treatments, respectively. Data are presented as mean ± SD (ns indicates no statistical significance; $^{*}P < 0.05$, $^{**}P < 0.01$, $^{***}P < 0.001$; $n = 3$).

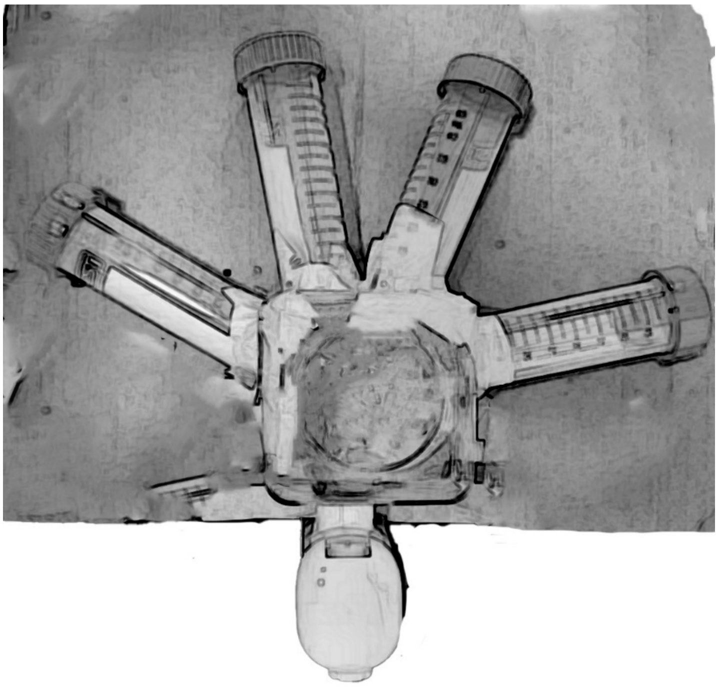

Figure S9. A self-made parallel high-throughput nebulization device suitable for multiple mice.

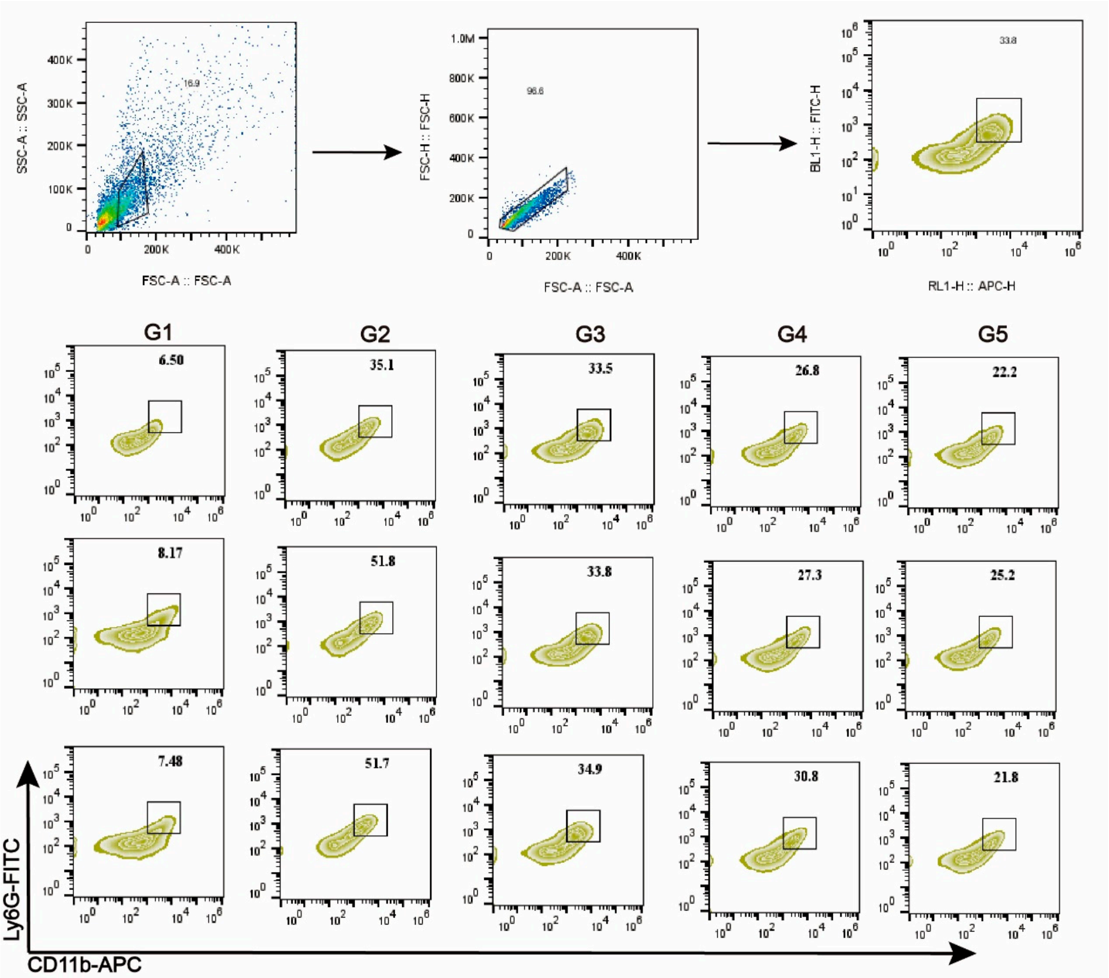

Figure S10. Gating strategy and flow cytometry plots for neutrophils in BALF.

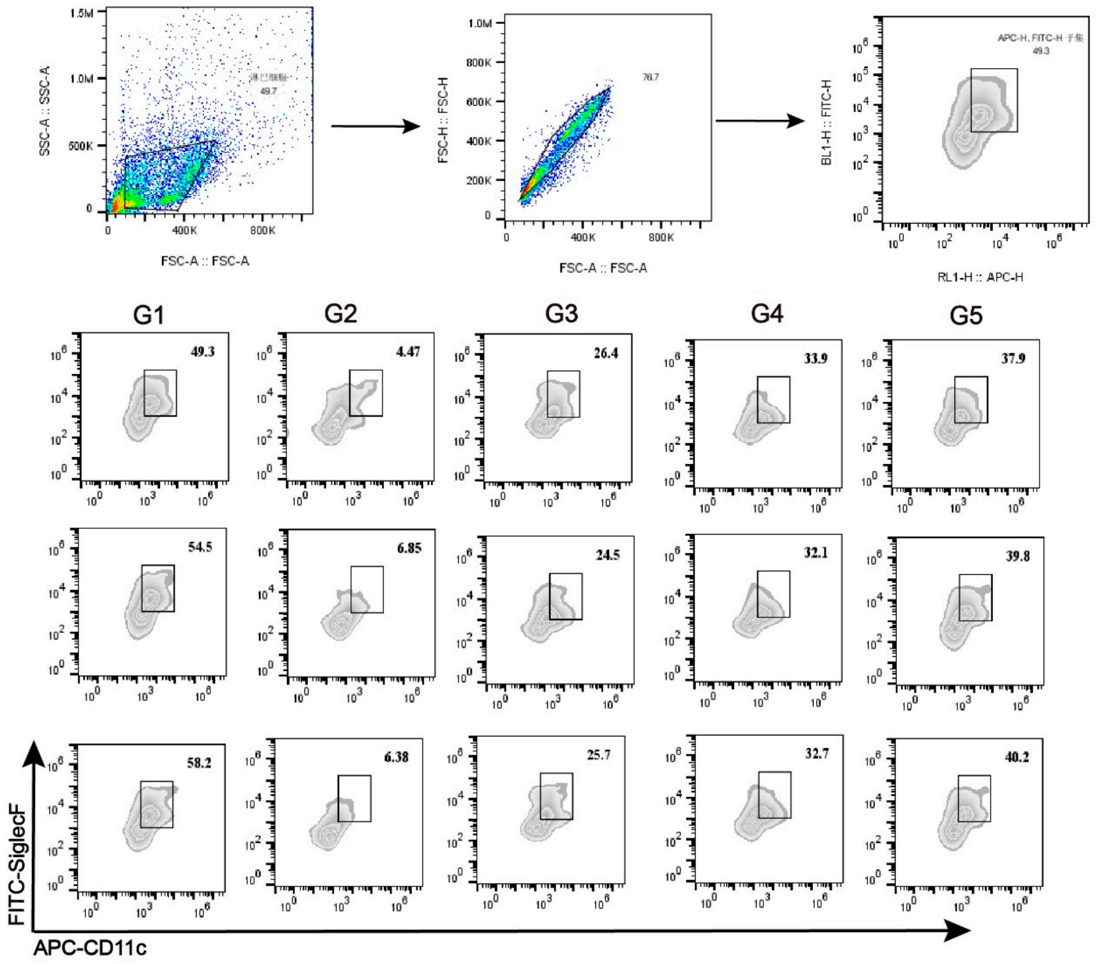

Figure S11. Gating strategy and flow cytometry plots for alveolar macrophages in BALF.

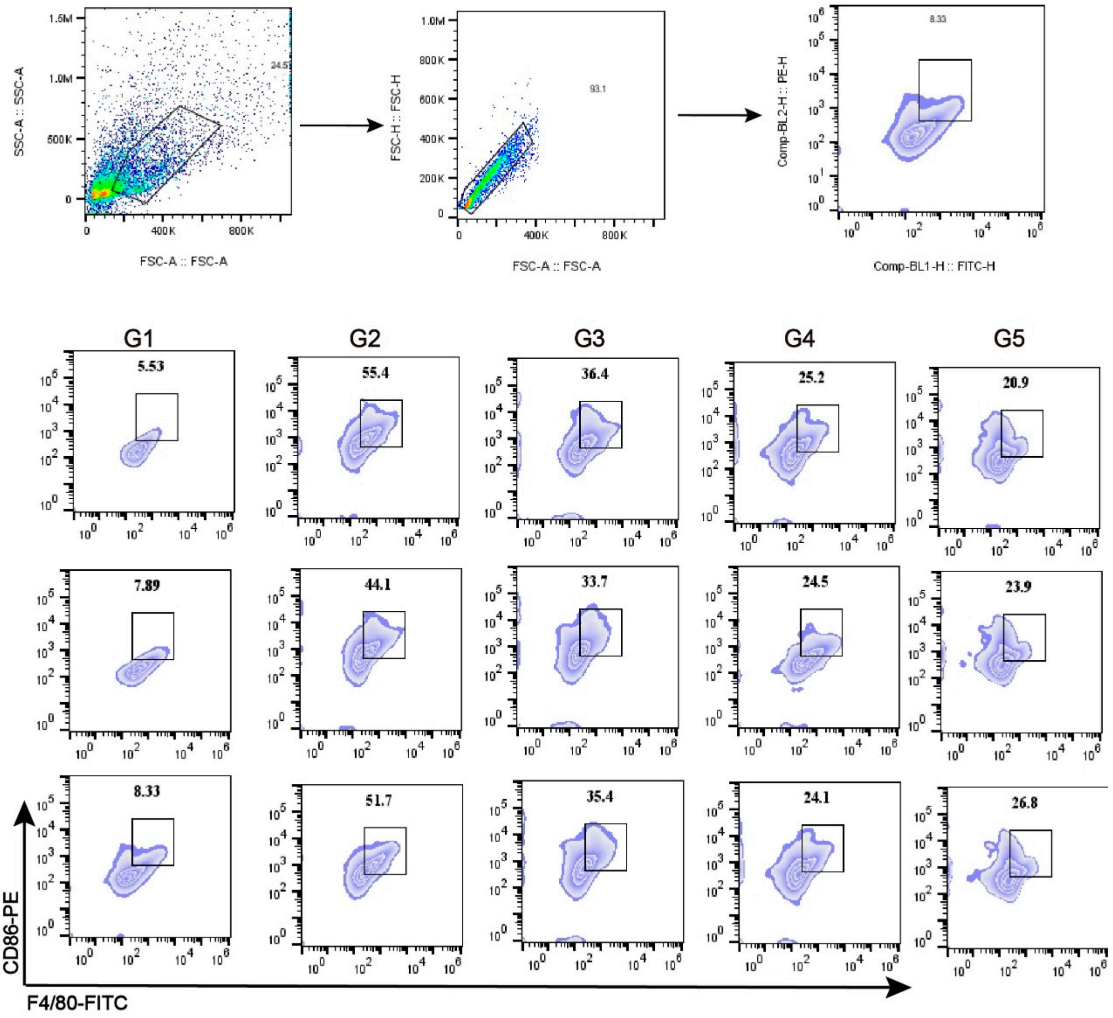

Figure S12. Gating strategy and flow cytometry plots for M1 macrophages in lung tissue.

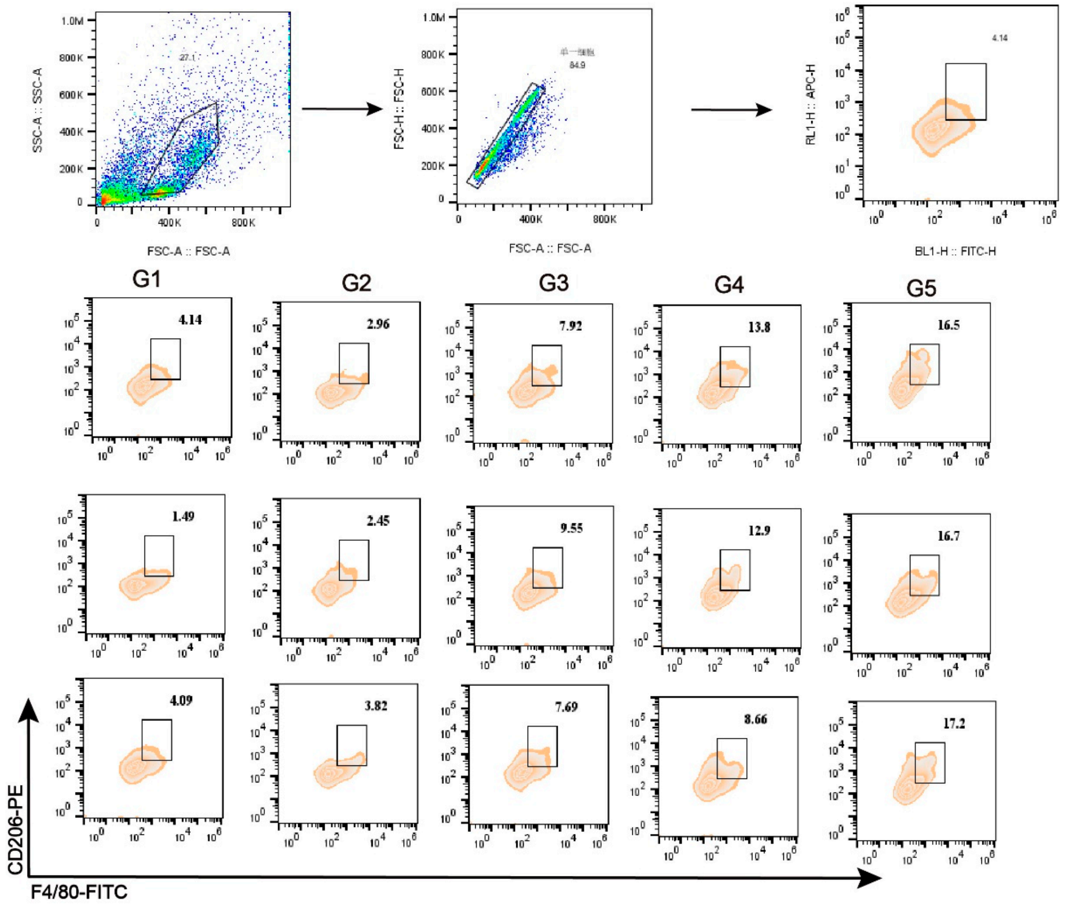

Figure S13. Gating strategy and flow cytometry plots for M2 macrophages in lung tissue.

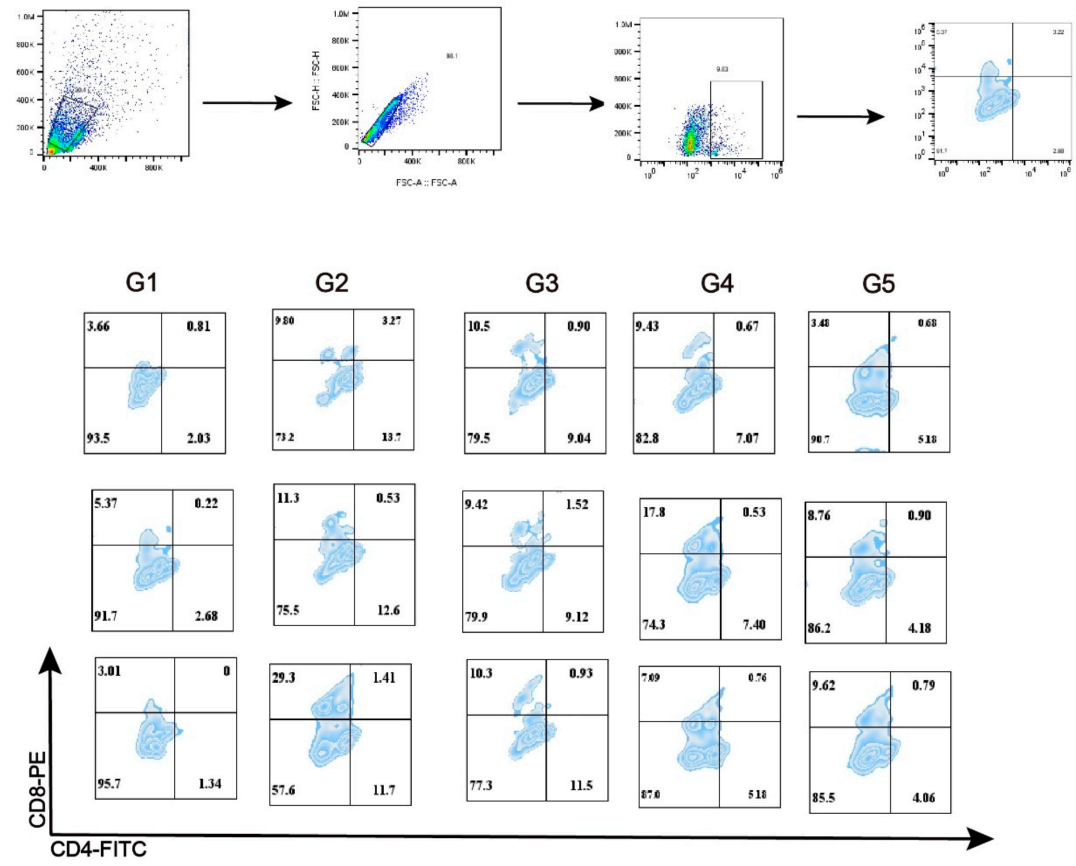

Figure S14. Gating strategy and flow cytometry plots for $CD4^+$ T cells and $CD8^+$ T cells in lung tissue.

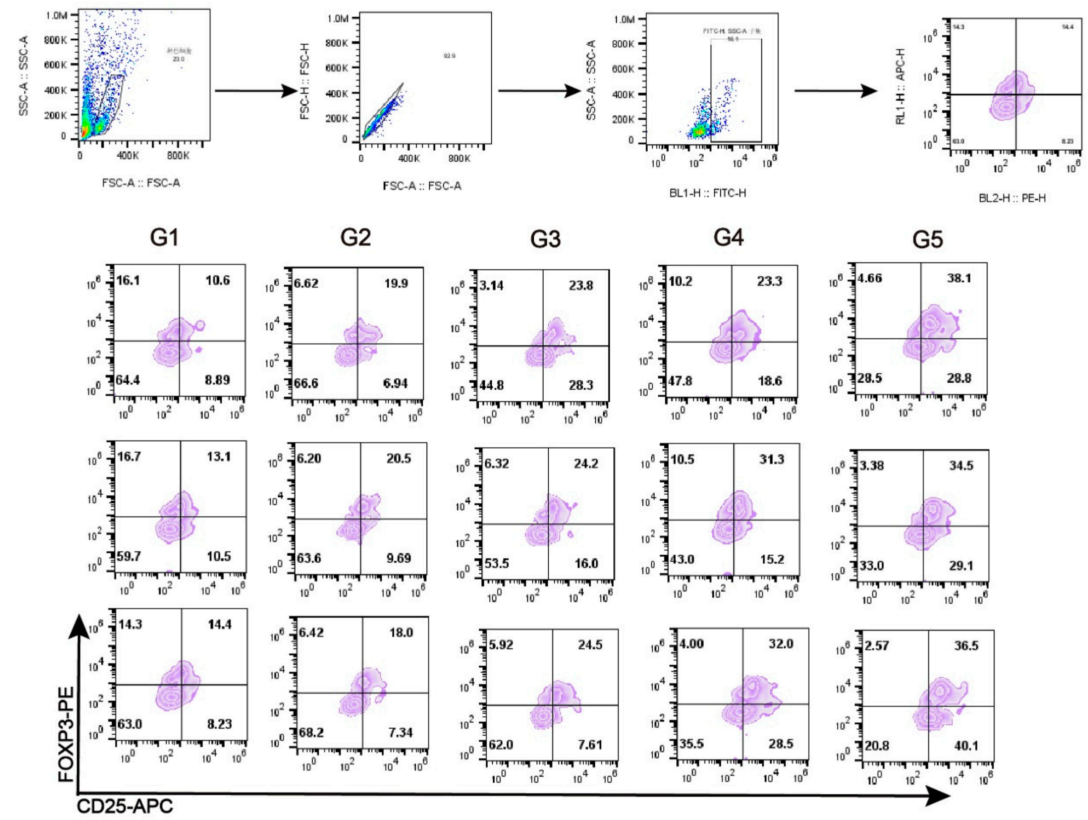

Figure S15. Gating strategy and flow cytometry plots for regulatory T cells (Tregs) in lung tissue.

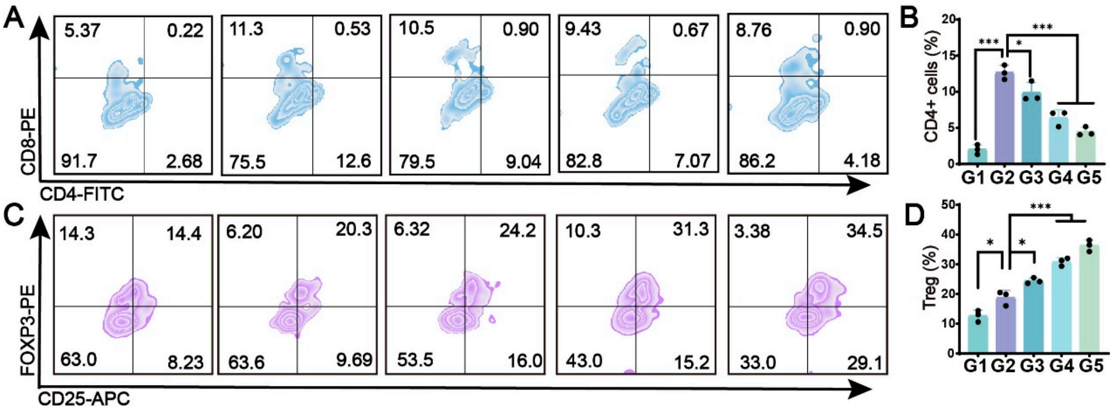

Figure S16. (A) shows representative flow cytometry scatter plots of CD4^{+} T cells and CD8^{+} T cells. (B) displays the quantitative statistical bar graph of the percentage of CD4^{+} T cells alone. (C)Flow cytometry plots of regulatory T cells (Tregs) in lung tissue. (D) Percentage distribution of regulatory T cells (Tregs) in lung tissue.

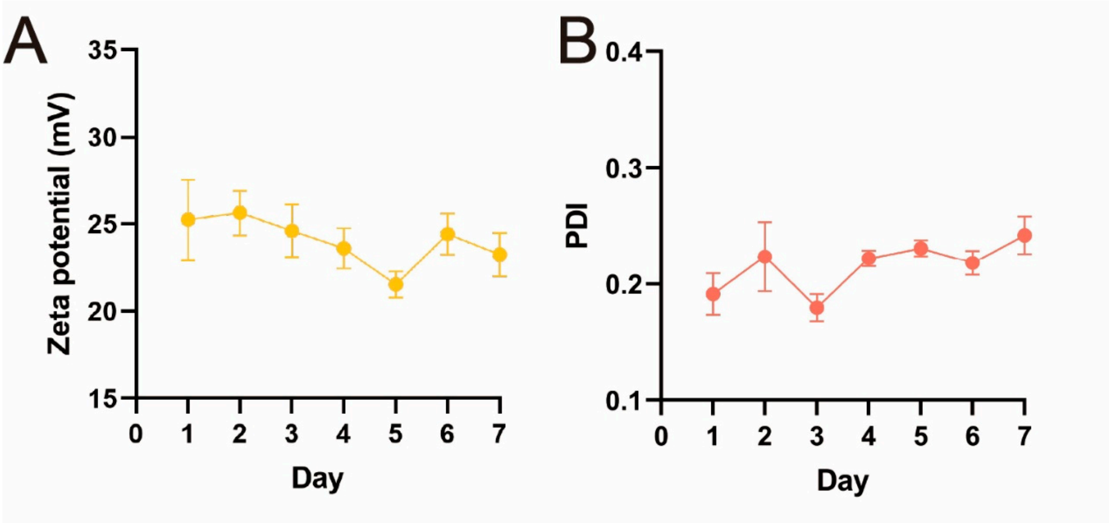

Figure S17. (A) Changes in zeta potential of HELNs at different storage days at 4°C. (B) Changes in polydispersity index (PDI) of HELNs at different storage days at 4°C.

Table S1. Flow antibody information

Antibody Name	Fluorescence Dyes	Cat	Clone
F4/80	FITC	123107	BM8
CD206	APC	141708	C068C2
CD86	PE	105007	CL-1
CD3	APC	100235	17A2
CD8	PE	100707	53-6.7
CD4	FITC	100405	GK1.5
FOXP3	PE	320007	150D
CD25	APC	102011	PC61
Ly6G	FITC	127605	1A8
SigleF	APC	155507	S17007L
CD11c	FITC	117305	N418
CD11b	APC	101211	M1/70
